# DAF-21/Hsp90 is required for *C*. *elegans* longevity by ensuring DAF-16/FOXO isoform A function

**DOI:** 10.1038/s41598-018-30592-6

**Published:** 2018-08-13

**Authors:** Milán Somogyvári, Eszter Gecse, Csaba Sőti

**Affiliations:** Department of Medical Chemistry, Semmelweis University, Budapest, Hungary

## Abstract

The FOXO transcription factor family is a conserved regulator of longevity and the downstream target of insulin/insulin-like signaling. In *Caenorhabditis elegans*, the FOXO ortholog DAF-16A and D/F isoforms extend lifespan in *daf*-*2* insulin-like receptor mutants. Here we identify the DAF-21/Hsp90 chaperone as a longevity regulator. We find that reducing DAF-21 capacity by *daf*-*21*(*RNAi*) initiated either at the beginning or at the end of larval development shortens wild-type lifespan. *daf*-*21* knockdown employed from the beginning of larval development also decreases longevity of *daf*-*2* mutant and *daf*-2 silenced nematodes. *daf*-*16* loss-of-function mitigates the lifespan shortening effect of *daf*-*21* silencing. We demonstrate that DAF-21 specifically promotes *daf*-*2* and heat-shock induced nuclear translocation of DAF-16A as well as the induction of DAF-16A-specific mRNAs, without affecting DAF-16D/F localization and transcriptional function. DAF-21 is dispensable for the stability and nuclear import of DAF-16A, excluding a chaperone-client interaction and suggesting that DAF-21 regulates DAF-16A activation upstream of its cellular traffic. Finally, we show a selective requirement for DAF-21 to extend lifespan of DAF-16A, but not DAF-16D/F, transgenic *daf*-*2* mutant strains. Our findings indicate a spatiotemporal determination of multiple DAF-21 roles in fertility, development and longevity and reveal an isoform-specific regulation of DAF-16 activity.

## Introduction

A conserved endocrine insulin/insulin-like nutrient signaling pathway (ILS) modulates longevity from yeast to mammals^1,2^. The Forkhead box O (FOXO) transcription factor family is the critical downstream mediator of the ILS pathway^3^. Activation of a Forkhead transcription factor consistently extends lifespan from yeast^4^ through worms^5,6^ to flies^7^. In mice, FOXO3 is required for dietary restriction induced longevity^8^ and loss of FOXO function appears to play a role in various age-related diseases including diabetes, cancer and atherosclerosis^9^. A potential role of FOXO in human longevity is corroborated by independent studies reporting the enrichment of certain FOXO1 and FOXO3a polymorphisms in nonagenarians^10,11^.

Much of our knowledge on the regulation of ILS and FOXO function are obtained through studies in the roundworm *Caenorhabditis elegans*^1,2^. Here, under nutrient-rich conditions insulin-like peptides (ILPs) bind to the DAF-2 insulin-like receptor which in turn initiates a signal cascade going through the *C*. *elegans* phosphoinositide 3-kinase AGE-1, the serine-threonine kinases PDK-1, AKT-1 and AKT-2^12^. Phosphorylation of the single FOXO ortholog DAF-16 by AKT kinases in three conserved serine/threonine residues leads to its exclusion from the nucleus thereby preventing its transcriptional function. In low nutrient conditions DAF-16 translocates into the nucleus and activates DAF-16 dependent gene expression^13^. Besides the AKT-mediated inhibitory phosphorylations there are a number of mechanisms regulating DAF-16/FOXO function including activatory phosphorylations, redox modifications, ubiquitinylation and proteasomal degradation revealing a highly complex integration of various regulatory inputs at the level of DAF-16/FOXO protein which is not fully understood^14^.

The transcriptional output of activated DAF-16/FOXO is similarly abundant. Targets shared across species and tissues throughout evolution encompass genes involved in growth factor signaling, metabolism, resistance to various stresses, and proteostasis^15^, indicating a conserved general longevity promoting “core” gene set. Indeed, DAF-16/FOXO has an intimate connection to stress. It is both activated by, and confers tolerance to, various stresses^16^. Likewise, reduced ILS mutants not only exhibit increased lifespan but also extended healthspan with more robust cross-tolerance to stresses^17^. Moreover, the proposed role of DAF-16/FOXO in proteostasis is illustrated by the facts that it upregulates genes of both autophagy and proteasomal protein degradation, while downregulates those of protein synthesis^3,18^. Finally, DAF-16 acts in concert with the heat shock transcription factor HSF-1 to extend nematode lifespan in response to reduced ILS^19^, in part by a cooperative induction of the longevity promoting small heat shock protein genes^19^. The above-mentioned findings both illustrate an important role of DAF-16 in proteostasis and imply further crosstalks to finely tune the DAF-16 and HSF-1 dependent responses.

In contrast to the four mammalian FOXO genes originating from gene duplication, the single *C*. *elegans* DAF-16 gene gives rise to three groups of transcripts that are transcribed from different promoters^6,20^. Isoforms A, B and D/F/H share the same Forkhead domain but have distinct N-terminal domains^2^. Further studies consistently revealed that DAF-16A and DAF-16D/F are responsible for lifespan extension^20,21^, however, their relative contribution remains controversial. These findings add another layer and a yet unknown complexity to DAF-16 regulation.

The 90-kDa heat-shock protein (Hsp90) is an essential, evolutionarily conserved eukaryotic molecular chaperone^22^. It accounts for 1–2% of the total cellular proteins. In yeast, Hsp90 is the most connected protein, affecting the function of ~20% of the proteome^22^. In mammalian cells Hsp90 is essential for the folding and activity of several hundred thermodynamically unstable ‘client’ proteins, predominantly kinases and transcription factors mainly involved in cell signaling and proliferation^23^. A comprehensive list of Hsp90 clients can be found at the Picard laboratory homepage (www.picard.ch/downloads/Hsp90interactors.pdf). Although Hsp90 is a promising tumor target^24^ and is implicated in the evolution of new traits^22^, several pieces of evidence suggest it might play a role in aging. For example, others and we reported that Hsp90 also chaperones antiproliferative, differentiation promoting clients, such as the TGF-β receptor^25^ and PPARγ^26^. Moreover, Hsp90 also regulates the heat-shock response by releasing the heat shock factor HSF-1 upon binding to denatured proteins^27^ which may be utilized in age-related diseases of proteostasis and metabolism, such as diabetes^28^. Yet, the impact of Hsp90 on aging is largely unexplored.

Despite the plethora of information in yeast and mammals, little is known about the biological function and interactions of the sole *C*. *elegans* Hsp90 ortholog encoded by the *daf*-*21* gene. Previous work using a weak gain-of-function point mutant *daf*-*21*(*p673*) unraveled its role in chemosensory perception and dauer development through DAF-11^29,30^, and muscle homeostasis *via* UNC-45^31^. The loss of DAF-21 in embryos either by large deletion mutation in the *daf*-*21*(*nr2081*) strain^30^ or in the F1 progeny of *daf*-*21*(*RNAi*) treated worms^32^ induced embryonic and early larval lethality and revealed a conserved function of Hsp90 in cell division through chaperoning the WEE-1 kinase^32–34^. Perhaps due to the essential role of *daf*-*21* in embryogenesis, its role in *C*. *elegans* lifespan regulation is largely unexplored. In this study, using gene silencing from larval development we investigated the involvement of DAF-21 in wild-type and *daf*-*2* induced longevity and found its specific requirement for DAF-16 isoform A activity.

## Results

### *daf*-*21/hsp90* knockdown silences DAF-21 expression and induces the heat shock response

**Figure 1:**
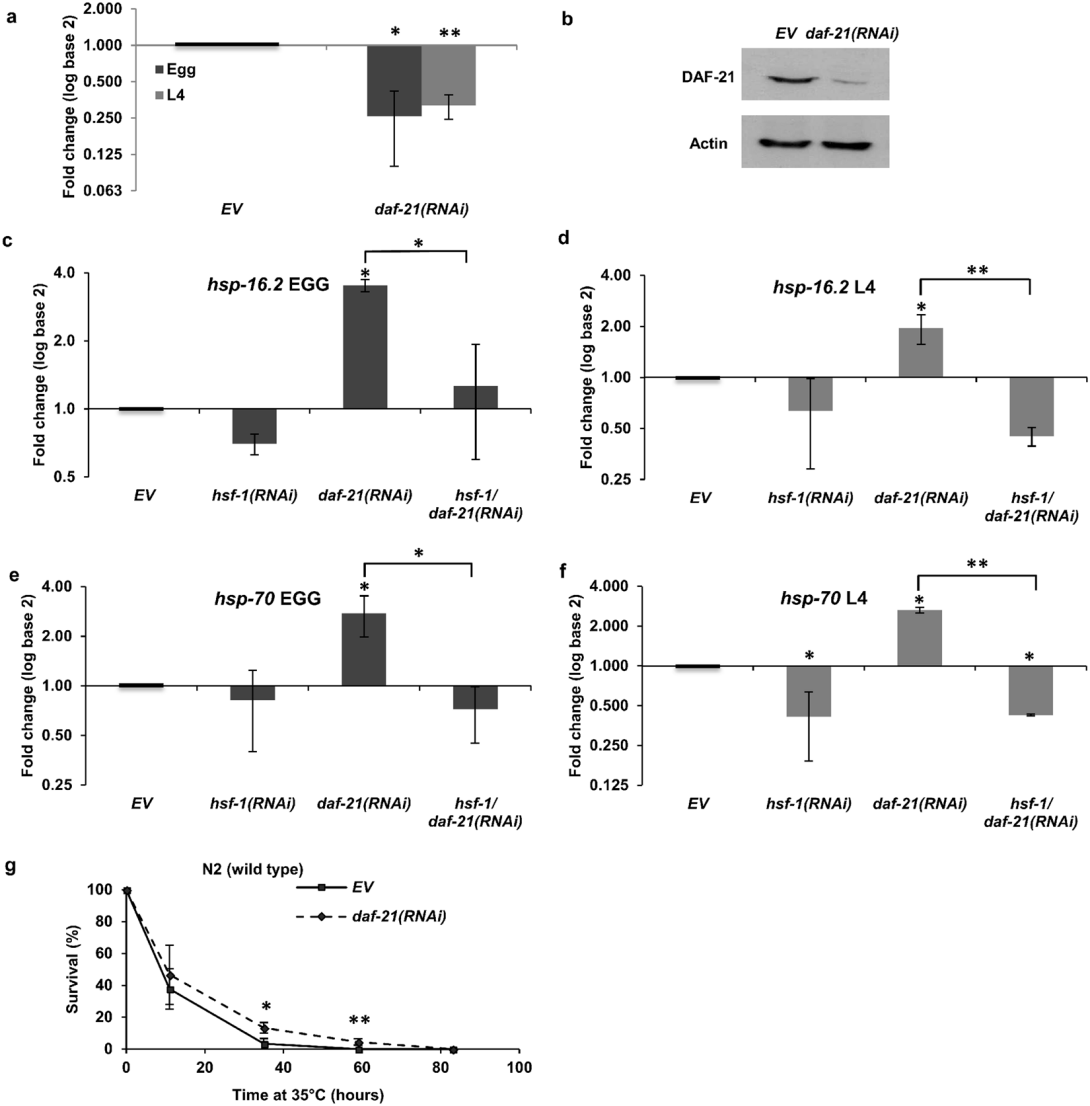
DAF-21/Hsp90 knockdown reduces *daf*-*21* mRNA and protein expression and induces the heat shock response. *daf*-*21*(*RNAi*) reduces *daf*-*21* mRNA (**a**) and protein (**b**) levels, respectively. Protein levels for DAF-21 and actin presented here were derived from the same membrane cut in two and incubated independently using their respective antibodies. Full-length blots are presented in Supplementary Fig. S1. *daf*-*21*(*RNAi*) employed either from hatching (**c**,**e**) or from the L4 stage (**d**,**f**) induces *hsp*-*16*.*2* (**c**,**d**) and *hsp*-*70* (*C12C8*.*1*) (**e**,**f**) mRNA levels at non-heat shock conditions in an *hsf*-*1* dependent manner and increases survival during heat shock (**g**). mRNA expression was assayed by qRT-PCR, normalized to *β*-*actin* mRNA and log2 transformed fold change values (mean ± SEM) were expressed relative to the respective EV control values of one. Please note that the asymmetric length of SEM error bars are a consequence of the logarithmic scale. Data shown are from three independent experiments. qRT-PCR statistics were analyzed by ANOVA and are given in Supplementary Table S3. Survival data were compared using Student’s t-test. EV: empty vector RNAi. *p < 0.05; **p < 0.01; ***p < 0.001.

To investigate the impact of reduced DAF-21 capacity on longevity we employed a *daf*-*21*(*RNAi*) construct described earlier^35^. In agreement with an essential role of Hsp90 in cell proliferation and development^22^ *daf*-*21*(*RNAi*) treatment of the parental F0 generation induced embryonic and early larval lethality in F1 in one^32^, and sterile F1 worms exhibiting developmental phenotypes in another study^35^. To investigate the impact of DAF-21 during larval development we started *daf*-*21* silencing from hatching. *daf*-*21*(*RNAi*) efficiently reduced *daf*-*21* mRNA and protein levels in young adults compared to empty vector (*EV*) control (Fig. 1a and b). Consistent with its reported role in vulval development and muscle function^35,36^, *daf*-*21*(*RNAi*) fed worms exhibited a protruding vulva in ~90% of the population and a mild hypomotility, but no other developmental phenotypes (Fig. S1). Likewise, *daf*-*21* silencing from hatching neither arrested nor significantly delayed development (Fig. S1). These observations indicate that *daf*-*21* knockdown employed from hatching does not induce larval lethality and developmental abnormalities^32^, except aberrant vulval morphogenesis during the L4 phase^37^.

To differentiate between the effects exerted by DAF-21 during larval development and adulthood, respectively, we also treated worms with *daf*-*21*(*RNAi*) from the midst of L4 stage. As expected, no protruding vulva phenotype was detected. Reflecting an essential role in oogenesis and embryonic development, *daf*-*21* silencing from hatching caused sterility accompanied by a lack of oocytes in the gonad, whereas that from the L4 stage caused reduced brood size and sometimes embryonic lethality (Supplementary Fig. S1d,e). *daf*-*21* knockdown employed from hatching or from the L4 stage similarly induced an *hsf*-*1* dependent *hsp*-*16*.*2* and *hsp*-*70* mRNA expression in young adults (Fig. 1c–f), consistent with a compensatory activation of the heat shock response upon the reduction of available DAF-21 protein^27^. We also observed a modest, but significant elevation of survival during heat shock in young adults fed by *daf*-*21*(*RNAi*) from hatching (Fig. 1g), reflecting an increased stress resistance associated with longevity and healthspan^38^. Hence, *daf*-*21*(*RNAi*), employed either during or after larval development is a safe approach to reduce DAF-21 capacity without compromising develoment and health.

### Reduction in DAF-21/Hsp90 capacity limits normal lifespan

**Figure 2:**
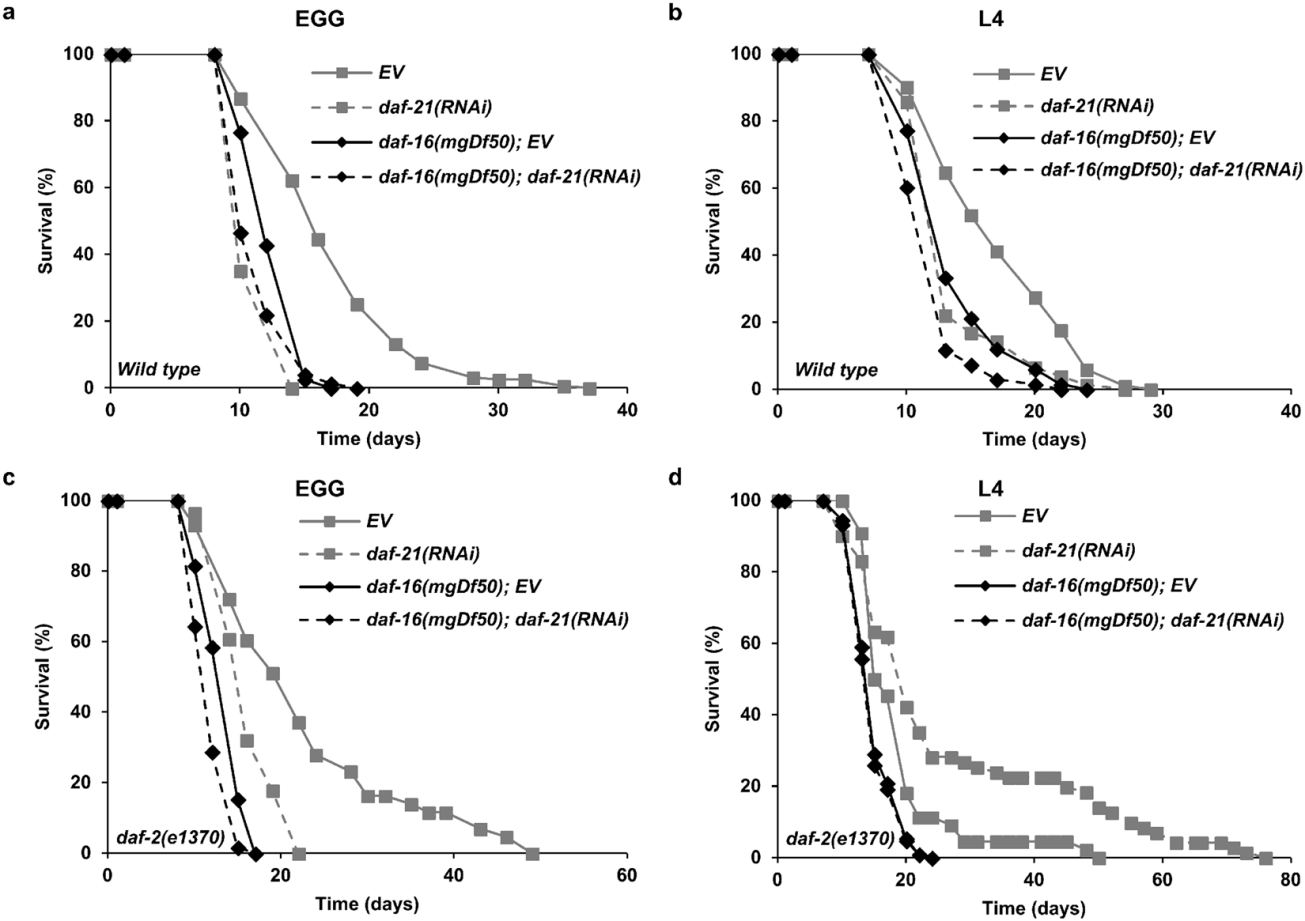
DAF-21/Hsp90 is required for normal lifespan and affects reduced ILS conferred longevity. *daf*-*21*(*RNAi*) employed either from hatching (**a**) or from the L4 stage (**b**) reduces wild-type lifespan to the level of the *daf*-*16*(*mgDf50*) mutant (p < 0.001 and p < 0.001 respectively). The *daf*-*2*(*e1370*) allele significantly increases the lifespan of worms with wild-type *daf*-*16* (p < 0.001) but not that of *daf*-*16*(*mgDf50*) mutants (p = 0.958). *daf*-*21*(*RNAi*) employed from hatching (**c**) reduces, whereas from the L4 stage (**d**) does not significantly affect the extended lifespan of *daf*-*2*(*e1370*) mutants (p = 0.001 vs. p = 0.076). Likewise, *daf*-*21*(*RNAi*) treatment from hatching reduces, but from the L4 stage does not affect the lifespan of *daf*-*2*(*e1370*); *daf*-*16*(*mgDf50*) double mutants (p < 0.001 and p = 0.435 in panel c and d, respectively). Lifespan assays were repeated three times. Survival curves were compared using the Kaplan-Meyer log rank test. Lifespan values are given in Supplementary Table S1. EV: empty vector RNAi.

Next, we addressed how reducing DAF-21 capacity starting at the beginning or at the end of larval development, respectively, might affect the natural lifespan of wild-type nematodes. First, we measured the lifespan of N2 worms fed by either empty vector or *daf*-*21*(*RNAi*) from hatching throughout the entire life. Our results show that *daf*-*21*(*RNAi*) shortened lifespan by ~27% compared to EV (Fig. 2a and Table S1). The reduction in lifespan caused by *daf*-*21*(*RNAi*) was less pronounced, but still significant, if RNAi treatment was administered from the L4 stage throughout adulthood (Fig. 2b). The *daf*-*21*(*p673*) gain-of-function allele appears to decrease lifespan mainly *via* bagging (death resulting from a deficiency in egg-laying and internal hatching of the progeny)^31^. We did not observe bagging and premature death in the *daf*-*21* silenced population. The comparable lifespan reduction by *daf*-*21* knockdown during and after development, respectively, suggests that *daf*-*21* affects longevity independent of egg laying and fertility (Supplementary Fig. S1d,e). The decreased lifespan, despite an increased heat shock response, which predicts and induces longevity^39,40^ might be the consequence of different longevity promoting mechanism(s) impaired in the absence of an optimal DAF-21 capacity.

### *daf*-*21/hsp90* is required from larval development for longevity conferred by reduced ILS

Increased longevity of animals with reduced ILS has been established as one of the most robust effects that prolong lifespan^5^. Therefore we compared the lifespan of *daf*-*2*(*e1370*) and *daf*-*2*(*e1370*); *daf*-*16*(*mgDf50*) single and double mutant animals, respectively, grown on plates with bacteria harboring empty vector or *daf*-*21*(*RNAi*). In accordance with the literature *daf*-*2* mutation caused an increase in lifespan compared to wild type which was entirely abrogated by *daf*-*16* loss of function (Fig. 2c and Table S1)^20,41,42^. *daf*-*21*(*RNAi*) treatment from hatching significantly reduced the lifespan of *daf*-*2*(*e1370*) worms showing a requirement for DAF-21 to fully manifest the increased longevity conferred by reduced ILS. The partial inhibitory effect of *daf*-*21*(*RNAi*) might be due to the unsuccessful penetration of dsRNA into neurons which do not express SID-1 protein^43^. To address this possibility, we used RNA interference to silence the expression of *daf*-*2* combined with *daf*-*21* knockdown. The lifespan of wild-type animals upon *daf*-*2*(*RNAi*)*/EV* showed a ~100% increase compared to empty vector (Supplementary Fig. S2 and Table S1) which was abolished in *daf*-*16*(*mu86*) mutant background. Again, we found a 20–50%, partial decrease of the lifespan of *daf*-*2* silenced nematodes upon *daf*-*21*(*RNAi*) treatment comparable to that observed in *daf*-*2* mutants, indicating a longevity supporting effect of DAF-21 in non-neuronal tissues.

Next, we determined the effect of *daf*-*21*(*RNAi*) employed from the L4 stage on the lifespan of *daf*-*2* mutants. In all 4 trials it extended the longevity of *daf*-*2*(*e1370*) animals, however, in two of them the effect was non-significant (Fig. 2d and Table S1). These observations indicate a longevity supporting effect of DAF-21 primarily during, but not after, development, in both wild-type and *daf*-*2* nematodes. We asked if *daf*-*21* affected the fertility of *daf*-*2* mutants. Consistent with earlier findings^44^ *daf*-*2* mutation delayed reproduction which was similarly abolished by *daf*-*21*(*RNAi*) employed either from hatching or from the L4 stage (Supplementary Fig. S1f,g). Thus, the differential effect of the *daf*-*21*(*RNAi*) treatment on *daf*-*2* lifespan appears to be independent of fertility.

In the majority of experiments, *daf*-*21*(*RNAi*) further shortened the lifespan of *daf*-*16* and *daf*-*2*; *daf*-*16* mutants, however, it was significantly less than its lifespan shortening effect in the strains harboring wild-type *daf*-*16* (Figs 2, S2 and S3 and Supplementary Table S1). Moreover, *daf*-*16* eliminated the longevity increasing tendency of *daf*-*21*(*RNAi*) in early adulthood of *daf*-*2* strains (Fig. 2d). Thus, it appears that the longevity action of DAF-21 might possess both a *daf*-*16* dependent as well as an independent component in both wild-type worms and those with reduced ILS.

### *daf*-*21/hsp90* knockdown in neurons promotes dauer development of wild type, that in non-neuronal tissues does not affect dauer formation of *daf*-*2* mutants

**Figure 3:**
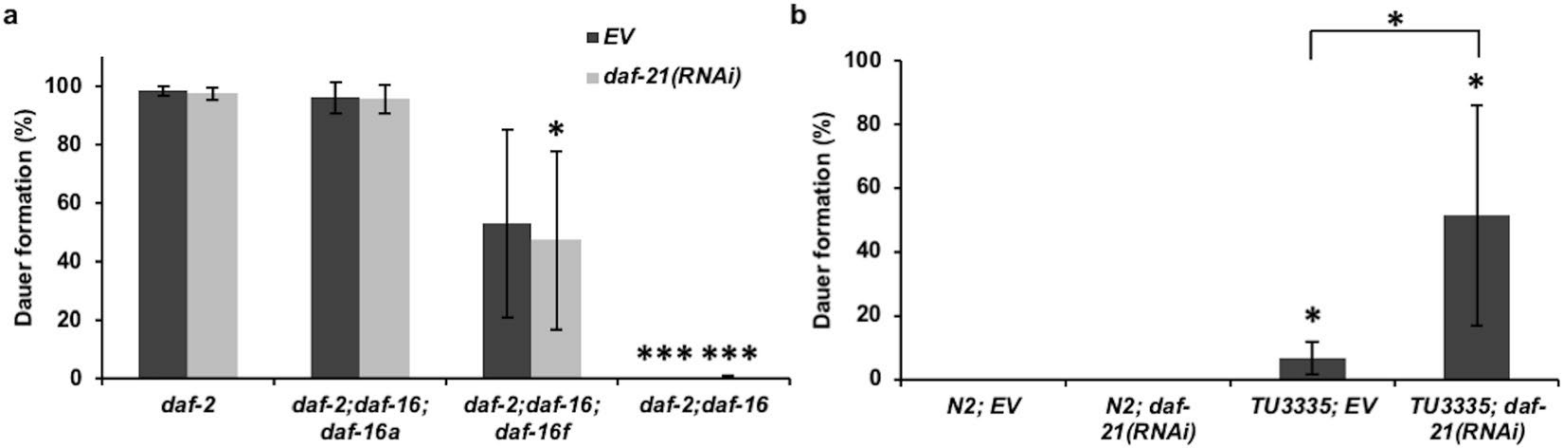
DAF-21/Hsp90 knockdown in non-neuronal cells does not affect dauer formation of wild type and *daf*-*2* nematodes. (**a**) Dauer formation of *daf*-*2*(*e1370*) worms at 25 °C. *daf*-*16a* is sufficient to mediate dauer formation, while *daf*-*16d/f* (p = 0.081 in EV and p = 0.037 in *daf*-*21*(*RNAi*) fed worms) exhibits reduced dauer formation. *daf*-*21*(*RNAi*) does not affect dauer formation when compared to EV. (**b**) Dauer formation of N2 and TU3335 whole body RNAi sensitive strains at 25 °C. *daf*-*21*(*RNAi*) only promotes dauer formation in TU3335 (p < 0.05) but not in wild type. Each experiment was repeated three times and expressed as mean ± SEM. Statistics were analyzed by ANOVA and are given in Supplementary Table S6. EV: empty vector RNAi. *p < 0.05; ***p < 0.001.

Nematodes with lowered ILS have a high tendency to initiate an alternative developmental pathway and form dauer larvae under stressful conditions. Likewise, the *daf*-*21*(*p673*) point mutant also promotes dauer formation in *C*. *elegans*^29^ by inhibiting the DAF-11 guanylcyclase in chemosensory neurons^30^. Hence, we investigated the interference between *daf*-*2* and *daf*-*21* in dauer development. As shown before^45^, *daf*-*2*(*e1370*) mutants grown on 25 °C almost exclusively turned into dauer larvae, while having a secondary mutation in *daf*-*16* abrogated this effect (Fig. 3a). In agreement with previous reports^20,21^ it appeared that *daf*-*16a* isoform *per se* is sufficient to manifest the dauer program while having only *daf*-*16d/f* resulted in a smaller rate of dauer formation. Dauer decision by lowered ILS is made at the L1 stage and requires predominantly neuronal *daf*-*2* and *daf*-*16*^44,46^. Consistent with this, silencing *daf*-*21* from hatching in non-neuronal cells did not affect dauer formation in any of the strains tested (Fig. 3a). Therefore, we employed the TU3335 strain that expresses SID-1 in each cell including neurons^47^ and confirmed and extended earlier findings about the neuronal requirement of sufficient DAF-21 function to bypass dauer arrest^29,30^ (Fig. 3b). Dauer decision by DAF-11 is also made at late L1 phase^48^, showing that *daf*-*21* is already silenced in (late) L1. The dauer arrest by neuronal *daf*-*21*(*RNAi*) and the embryonic/early larval lethality caused by the embryonic loss of DAF-21^30,32^ prevented us to test its impact on *daf*-*2* induced dauer formation. However, our findings together with the lifespan data indicate that DAF-21 in peripheral tissues affects wild-type and *daf*-*2* longevity that is spatiotemporally separated from its effect on development.

### DAF-21/Hsp90 facilitates DAF-16A nuclear translocation

**Figure 4:**
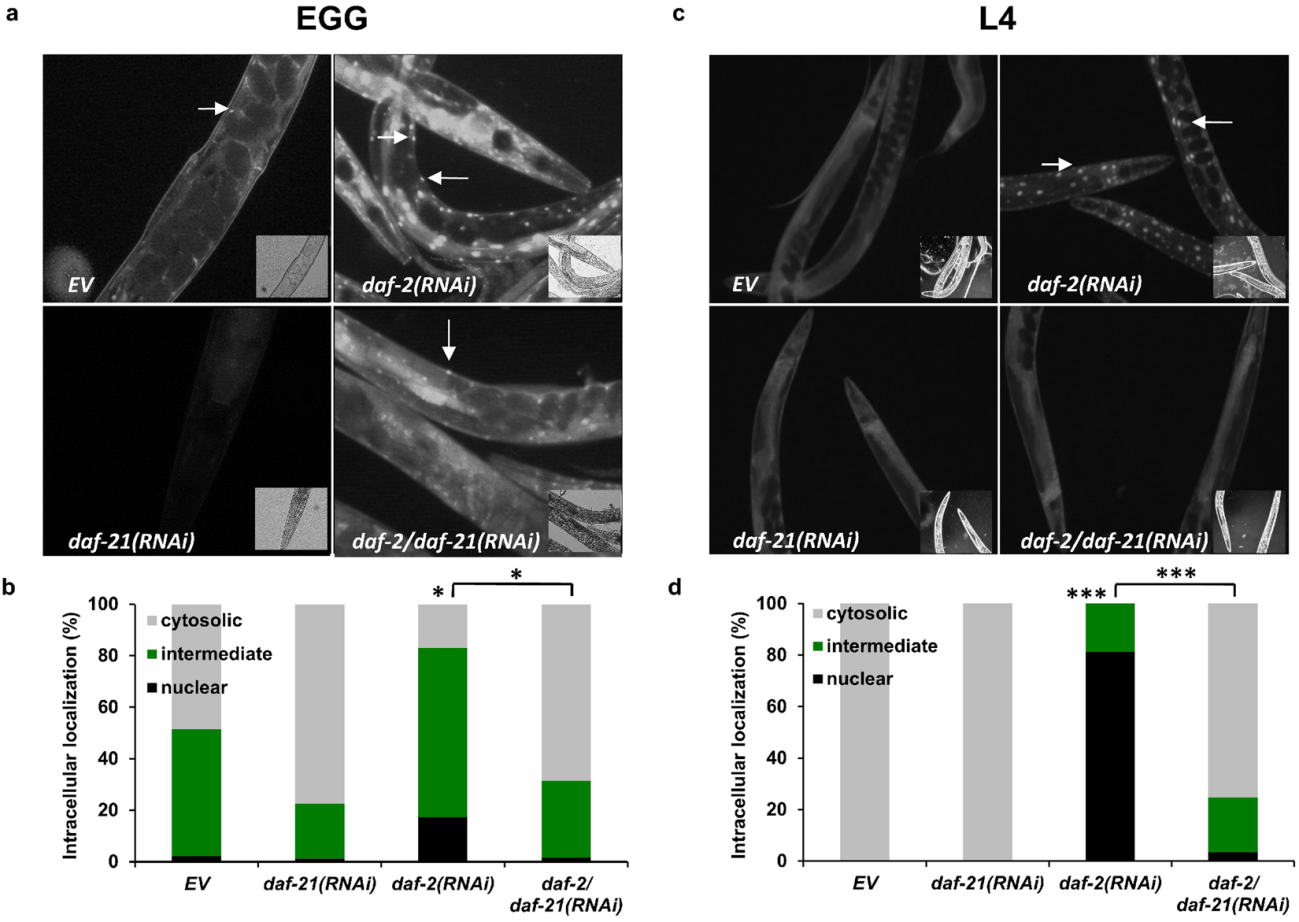
DAF-21/Hsp90 is required for *daf*-*2* induced DAF-16A/B nuclear translocation. Representative epifluorescence microscopic images showing the *daf*-*2*(*RNAi*) induced nuclear translocation of DAF-16A/B::GFP inhibited by *daf*-*21*(*RNAi*) treatment employed either from hatching (**a**) or from the L4 stage. (**c**) White arrows indicate nuclearly localized GFP. Quantification (mean ± SEM) of DAF-16A/B::GFP localization from three independent experiments, each experiment using 30 animals per condition (**b** and **d**). Cytosolic refers to animals without nuclearly localized GFP signal, intermediate refers to animals with nuclear and cytosolic GFP and nuclear refers to animals with solely nuclear GFP signal. Microscopic images are representatives of 3 independent experiments. Statistics were analyzed by ANOVA and are given in Supplementary Table S4. EV: empty vector RNAi. *p < 0.05; ***p < 0.001.

The fact that the impact of DAF-21 on *daf*-*2* lifespan is dependent on DAF-16 suggested a functional link between DAF-21 and DAF-16. Hence, we asked how *daf*-*21* knockdown affects DAF-16 function. An important step in DAF-16 activity in response to lowered ILS, as well as stresses including heat shock, is its nuclear translocation^49^. First, we monitored the intracellular localization of DAF-16 in the TJ356 strain containing a *daf*-*16a/b::GFP* transgene^16^ in response to *daf*-*2* and *daf*-*21* knockdown employed from hatching. To ensure similar RNAi dosages, animals were fed with bacteria containing empty vector (EV), *daf*-*2*(*RNAi*)*/EV*, *daf*-*21*(*RNAi*)*/EV* and *daf*-*2*(*RNAi*)*/daf*-*21*(*RNAi*) in 1:1 ratios. In *daf*-*2* silenced nematodes a large proportion of DAF-16A/B was localized in the nuclei of both intestinal as well as muscle cells. We found that silencing *daf*-*21* partially inhibited the *daf*-*2* knockdown-induced nuclear translocation of DAF-16A/B::GFP (Fig. 4a and b). This result indicates that DAF-21 facilitates DAF-16 translocation in response to lowered ILS. To address if this phenomenon might be the consequence of a process altered during development or it is present in adulthood, we repeated this measurement by placing L4 larvae on RNAi plates. The previously observed increase in DAF-16 nuclear localization upon *daf*-*2*(*RNAi*) was confirmed under these conditions as well as *daf*-*21*(*RNAi*)‘s ability to inhibit such translocation (Fig. 4c and d).

**Figure 5:**
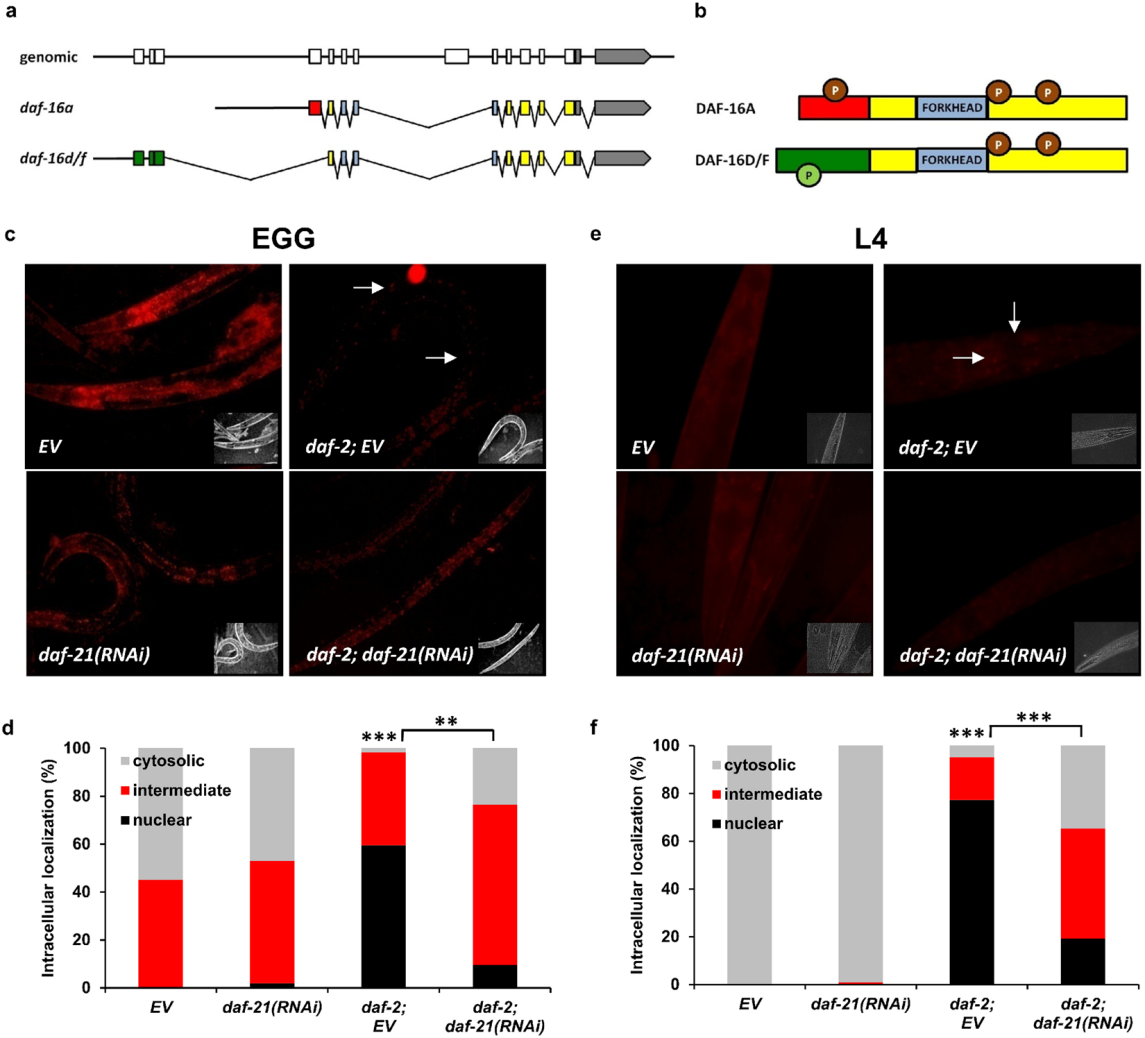
DAF-21/Hsp90 selectively regulates DAF-16A nuclear translocation. Schematic representation of the transcript (**a**) and protein (**b**) structures of the *daf*-*16a* and *daf*-*16d/f* isoforms. Coding regions are marked as colored boxes, noncoding regions as lines and the 3′ untranslated regions as grey boxes. Circles with “P” represent sites of phosphorylation. Please note their different N-terminal sequences as well as the different N-terminal consensus phosphorylation sites (indicated in different colors). (**c**,**e**) Representative epifluorescence microscopic images showing the *daf*-*2*(*e1370*) mutation induced nuclear translocation of DAF-16A::RFP inhibited by *daf*-*21*(*RNAi*) treatment employed either from hatching (**c**) or from the L4 stage. (**e**) White arrows indicate nuclearly localized RFP. Quantification (mean ± SEM) of DAF-16A::RFP localization from three independent experiments, each experiment using 30 animals per condition (**d** and **f**). Cytosolic refers to animals without nuclearly localized RFP signal, intermediate refers to animals with nuclear and cytosolic RFP and nuclear refers to animals with solely nuclear RFP signal. Microscopic images are representatives of 3 independent experiments. Statistics were analyzed by ANOVA and are given in Supplementary Table S4. EV: empty vector RNAi. n.s.: non-significant; *p < 0.05; **p < 0.01; ***p < 0.001.

We asked whether Hsp90 might act on DAF-16 function in an isoform-specific manner. Two out of the three DAF-16 isoform groups, A and D/F/H, are targeted by ILS in longevity regulation^6,20,21,50,51^. Therefore, we employed two strains that express different fluorescently tagged DAF-16 isoforms in a *daf*-*16*(*mgDf50*) null mutant background: *daf*-*16a::rfp* and *daf*-*16d/f::gfp* and their respective *daf*-*2*(*e1370*) variants^20^. The transcripts and proteins structures of the two isoforms are depicted in Fig. 5a and b. RNAi treatment was employed from hatching, and DAF-16 localizaton was visualized on day 1 of adulthood. Consistent with previous reports DAF-16A::RFP showed an explicit nuclear localization in both intestinal and muscle cells while DAF-16D/F::GFP remained largely cytosolic in response to *daf*-*2*(*e1370*) compared to the respective strains with intact *daf*-*2* alleles (Figs 5c,d and S6)^20,49^ regardless of growing temperature. *daf*-*21* knockdown, in accordance with its effect on DAF-16A/B::GFP, inhibited the translocation of DAF-16A::RFP, while did not affect the localization of DAF-16D/F::GFP (Figs 4a,b, 5c,d and S6). To gain an independent insight on the translocation of DAF-16 isoforms, we employed heat stress, during which both isoforms have been shown to enter the nucleus^20,49^. Indeed, heat shock induced a predominantly nuclear localization of DAF-16A/B::GFP, DAF-16A::RFP and DAF-16D/F::GFP (Supplementary Figs S4, S5 and S6). However, in response to *daf*-*21*(*RNAi*) DAF-16D/F::GFP was still nuclear, while the nuclear localization of both DAF-16A/B::GFP and DAF-16A::RFP were abolished (Supplementary Figs S4, S5 and S6). We obtained similar results by RNAi treatment from L4 larval stage, i.e. *daf*-*21* silencing inhibited both heat-shock and *daf*-*2* mutation induced DAF-16A nuclear translocation (Figs 5e,f and S5). These results besides illuminating a differential regulation of the DAF-16A and D/F isoforms, strongly support a specific and uniform requirement of DAF-21 for DAF-16A nuclear translocation under low nutrient and heat stress conditions, respectively.

### DAF-21/Hsp90 is required for DAF-16A dependent transcriptional function

**Figure 6:**
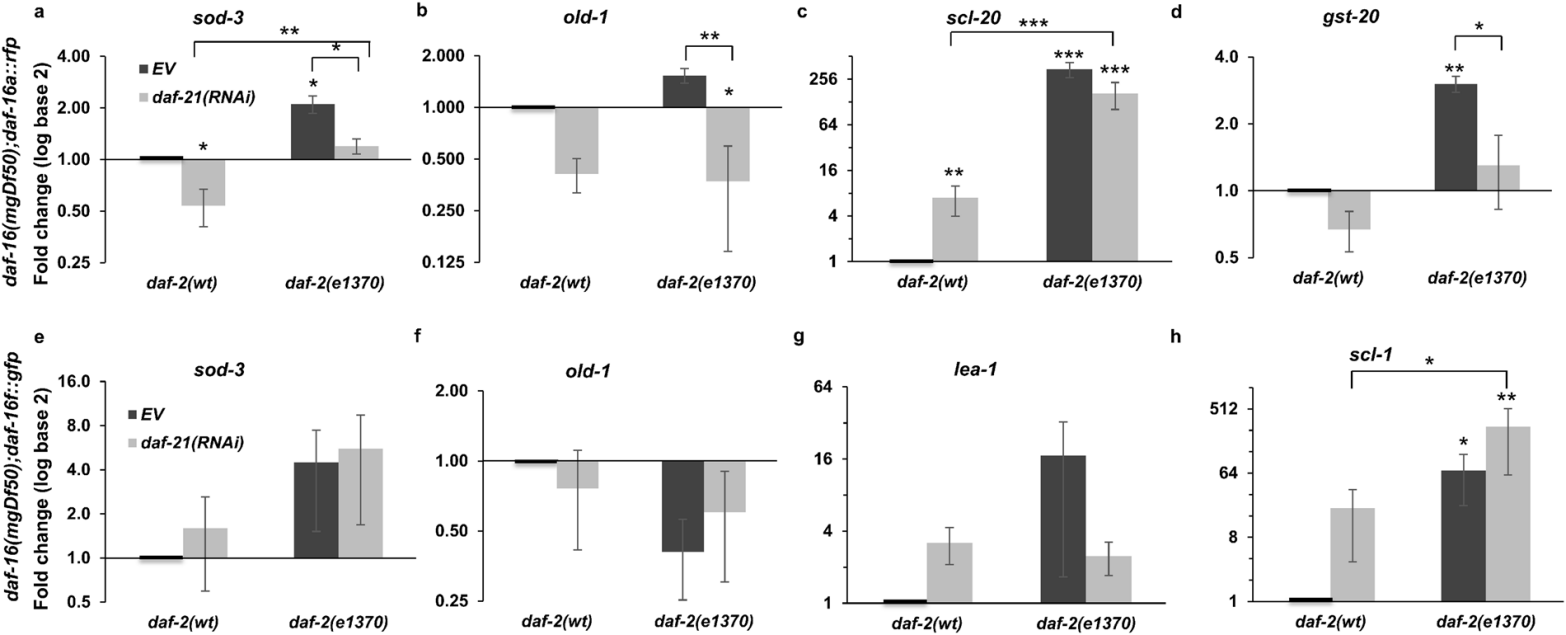
DAF-21/Hsp90 selectively regulates daf-16 dependent target gene expression in the DAF-16A single isoform transgenic background. (**a**–**d**) Effect of *daf*-*21*(*RNAi*) on *sod*-*3* (**a**) *old*-*1* (**b**) and the DAF-16A specific *scl*-*20* (**c**) and *gst*-*20* (**d**) mRNA levels in *daf*-*16*(*mgDf50*); *daf*-*16a::rfp* worms and its *daf*-*2*(*e1370*) derivative. (**e**–**h**) Effect of *daf*-*21*(*RNAi*) on *sod*-*3* (**e**) *old*-*1* (**f**) and the DAF-16D/F specific *lea*-*1* (**g**) and *scl*-*1* (**h**) mRNA levels in *daf*-*16*(*mgDf50*); *daf*-*16d/f::gfp* worms and its *daf*-*2*(*e1370*) derivative. Nematodes were fed by EV or *daf*-*21*(*RNAi*) from hatching. mRNA expression was assayed by qRT-PCR, normalized to *β*-*actin* mRNA and log2 transformed fold change values (mean ± SEM) were expressed relative to the respective EV control values of one. Data shown are from three independent experiments. qRT-PCR statistics were analyzed by ANOVA and are given in Supplementary Table S3. EV: empty vector RNAi. *p < 0.05; **p < 0.01; ***p < 0.001.

Next we studied how *daf*-*21* silencing affects the expression of various DAF-16 target genes. We looked into the expression of genes that are thought to be regulated by both isoforms (*sod*-*3* and *old*-*1*), some that are reportedly DAF-16A-regulated (*scl*-*20* and *gst*-*20*) and genes that are the targets of DAF-16 isoform F (*lea*-*1*, *scl*-*1*, *col*-*183*, *R05D8*.*7*). In the first series of experiments, in order to clearly isolate the undesired cross-talk from the other DAF-16 isoforms, we took use of the above *daf*-*16a::rfp* and *daf*-*16d/f::gfp* strains. The induction of *sod*-*3* and *old*-*1* mRNA expression – caused by the *daf*-*2*(*e1370*) mutation – were both diminished by *daf*-*21*(*RNAi*) in the *daf*-*16a::rfp* strain (Fig. 6a and b), while they were unaffected in the *daf*-*16d/f::GFP* strain (Fig. 6e and f). The *daf*-*21*-dependent nature of *sod*-*3* and *old*-*1* induction was reinforced by using *daf*-*2*(*RNAi*) on wild-type animals (Supplementary Fig. S7). Moreover, *old*-*1* was only upregulated in the *daf*-*2*(*e1370*) mutant variant of the *daf*-*16a::rfp* strain (Fig. 6b and f). These results corroborate previous data on *sod*-*3*^21^ and propose *old*-*1* as a preferential DAF-16A target. We also selected *scl*-*20* and *gst*-*20*, identified as specific DAF-16A targets^21^. *scl*-*20* encodes a putative p53 target that functions to regulate both lifespan and tumor cell proliferation^52^. *gst*-*20* is an ortholog of human hematopoietic prostaglandin D synthase and is involved in the *daf*-*2* induced, diet dependent extension of adult lifespan^21^. Our experiments confirmed the upregulation of both genes by the *daf*-*2*(*e1370*) allele in the *daf*-*16a::rfp* strain and showed an efficient inhibition of *gst*-*20* expression by *daf*-*21*(*RNAi*) (Fig. 6c and d). To address the influence of DAF-21 on the transcriptional activity of the DAF-16D/F isoform, *lea*-*1* and *scl*-*1* were selected for our purposes as selective targets^20,21^. *lea*-*1* encodes a protein that is predicted to be hydrophilic and heat-resistant, and that might participate in anhydrobiosis^53^, while *scl*-*1* encodes a predicted secretory protein that is a member of the cysteine-rich secretory protein (CRISP) family^54^. In contrast to DAF-16A target genes, these two transcriptional targets in a *daf*-*16d/f::GFP* transgenic background were not inhibited by *daf*-*21*(*RNAi*) (Fig. 6g and h).

**Figure 7:**
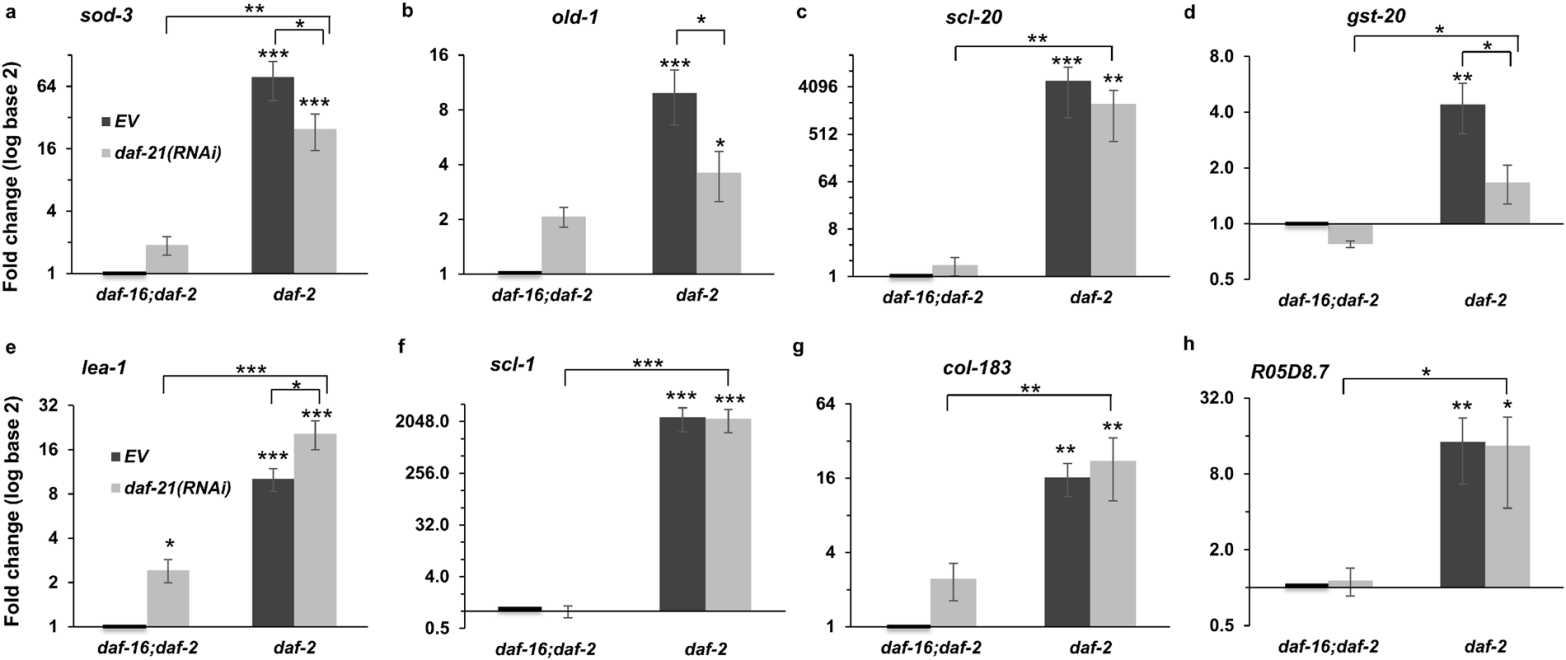
DAF-21/Hsp90 is specifically required for the expression of DAF-16A dependent target genes in wild-type daf-16 background. (**a**–**d**) Effect of *daf*-*21*(*RNAi*) on *sod*-*3* (**a**) *old*-*1* (**b**) and the DAF-16A specific *gst*-*20* (**c**) and *scl*-*20* (**d**) mRNA levels. (**e**–**h**) Effect of *daf*-*21*(*RNAi*) on the DAF-16D/F specific *lea*-*1* (**e**) *scl*-*1* (**f**), *col*-*183* (**g**) and *R05D8*.*7* (**h**) mRNA levels. *daf*-*2*(*e1370*) and *daf*-*2*(*e1370*); *daf*-*16*(*mgDf50*) double mutant worms were fed by *EV* or *daf*-*21*(*RNAi*) from the L4 stage. mRNA expression was assayed by qRT-PCR, normalized to *β*-*actin* mRNA and log2 transformed fold change values (mean ± SEM) were expressed relative to the respective EV control values of one. Data shown are from three independent experiments. qRT-PCR statistics were analyzed by ANOVA and are given in Supplementary Table S3. EV: empty vector RNAi. *p < 0.05; **p < 0.01; ***p < 0.001.

To further examine a potential isoform-specific regulation we measured the expression of DAF-16A and DAF-16D/F isoform specific targets in a wild-type and mutant *daf*-*16* background in response to *daf*-*21* silencing from the L4 stage. Comparing the mRNA expression in a *daf*-*2*(*e1370*) and *daf*-*16*(*mgDf50*); *daf*-*2*(*e1370*) strains, respectively, showed that the DAF-16A specific *sod*-*3*, *old*-*1*, *gst*-*20* and *scl*-*20* were induced by the *daf*-*2* mutation in a *daf*-*16* dependent manner and their expression was inhibited by silencing *daf*-*21* with the exception of *scl*-*20* (Fig. 7a–d). DAF-16D/F target genes were supplemented by two additional DAF-16D/F specific targets: *col*-*183*, a predicted structural constituent of cuticle^55^ and *R05D8*.*7*, an ortholog of human hydroxysteroid 17-beta dehydrogenase 14 involved in embryonic development^56^. We found that all mRNAs were efficiently induced in the *daf*-*2*(*e1370*) mutant but not inhibited by *daf*-*21*(*RNAi*) (Fig. 7e–h). Some of the findings were confirmed by using an independent *daf*-*21*(*RNAi*) sequence^35^ (Supplementary Fig. S8). Thus, our findings provide compelling evidence that DAF-21 specifically regulates the transcriptional activity of DAF-16A.

### DAF-21/Hsp90 is not necessary for DAF-16A stability and acts upstream of its nuclear import

Mammalian Hsp90 is a specific chaperone that stabilizes the conformation of a large number of client proteins including various transcription factors^22^. If Hsp90 function is compromised, destabilized clients are polyubiquitinylated by ubiquitin conjugating enzyme complexes and subsequently degraded by the proteasome^57^. Several E3 ubiquitin ligases target mammalian FOXOs to degradation^18^. In *C*. *elegans*, the null mutation of the RLE-1 E3 ubiquitin ligase has been shown to lead to DAF-16 protein stabilization and DAF-16 dependent lifespan extension^58^. We reasoned if DAF-21 stabilized DAF-16 conformation, then reduced DAF-21 capacity would result in DAF-16 aggregation and disrupt lifespan extension in *rle*-*1* mutants. Although we did not detect a substantial increase of DAF-16A::RFP in *rle*-*1* background, *daf*-*21*(*RNAi*) neither interfered with DAF-16 distribution nor caused DAF-16 aggregation (Supplementary Fig. S9). This might be due to the action of other, yet unidentified ubiquitin ligase(s). However, DAF-21 did not influence the turnover of DAF-16 protein: we neither observed a decrease in the quantity of various fluorescently tagged DAF-16A proteins (see Figs 4, 5, S4, S5 and S9) nor a compensatory upregulation *of daf*-*16a* mRNA (Supplementary Fig. S9) upon *daf*-*21* knockdown. Likewise, *rle*-*1* induced lifespan extension of the strain expressing solely the DAF-16A::RFP isoform still persisted in the absence of DAF-21 (Supplementary Fig. S9) indicating that the stabilization of a functional DAF-16 protein does not require DAF-21, although a DAF-16 independent effect of *rle*-*1* on lifespan is also plausible. In support of DAF-16 conformational stability, contrary to the detrimental effect of proteotoxic stresses such as heat shock on Hsp90 clients^23,26^, DAF-16 is activated during heat shock^16,49^ (and data presented herein). Thus, DAF-16 is unlikely to be a DAF-21 client.

Similarly to mammalian cells, when ILS signaling is ample, AKT-1 and AKT-2 kinases phosphorylate DAF-16/FOXO which prevents its accumulation in nuclei by anchoring it to cytosolic 14-3-3 scaffold proteins^49^. In response to reduced ILS, DAF-16 translocates into the nucleus. To assess if DAF-21 acts upstream or at the level of DAF-16 nuclear traffic, we employed a strain harboring the *daf*-*16a*^*AM*^*::gfp* (AM: “AKT site mutant”) transgene, in which serines and threonines in the AKT phosphorylation sites were changed to alanines^49^. In agreement with previous work^49^, DAF-16A^AM^::GFP was nuclearly localized in animals possessing wild-type *daf*-*2* (Supplementary Fig. S10). If DAF-21 was necessary for DAF-16A to achieve its native functional conformation, then reducing DAF-21 capacity would result in an unstable DAF-16A^AM^::GFP unable to enter the nucleus and would be degraded. This was not the case, because neither the quantity, nor the localization of DAF-16A^AM^::GFP was modified by *daf*-*21*(*RNAi*) (Supplementary Fig. S10), providing further evidence for the conformational independence of DAF-16A from DAF-21 and indicating that the nuclear import of unphosphorylated DAF-16A does not require DAF-21. Also, these findings made unlikely a DAF-21-dependent inhibition of nuclear export of DAF-16A. Instead, DAF-21 appears to influence DAF-16A activation upstream of its nuclear traffic. This result is supported by our measurements of DAF-16A-specific target genes in the DAF-16A^AM^ strain. Of the previously examined four specific DAF-16A targets only *scl*-*20* and *sod*-*3* were induced in the DAF-16A^AM^ strain when compared to the *daf*-*16*(*mu86*) mutant (Supplementary Fig. S10), although only *scl-20* reached the level of statistical significance. However, their expression was not altered by *daf*-*21*(*RNAi*), suggesting that nuclear DAF-16 is able to exert a partial transcriptional activity which is independent of DAF-21. These findings corroborate the idea that DAF-21 acts upstream of DAF-16A nuclear traffic.

### DAF-21/Hsp90 ensures *daf*-*16a* dependent longevity

**Figure 8:**
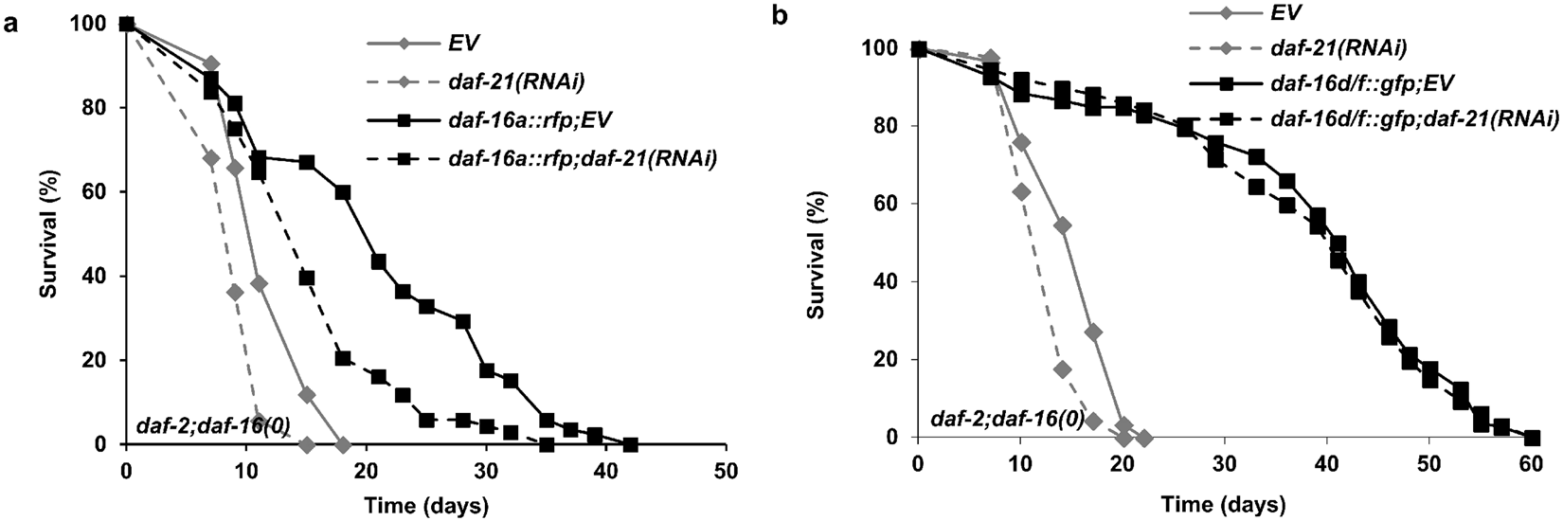
DAF-21/Hsp90 is required for DAF-16A, but not DAF-16D/F mediated lifespan extension. (**a**) The *daf*-*2*(*e1370*) allele increases lifespan in *daf*-*16*(*mgDf50*); *daf*-*16a::rfp* worms (p < 0.001). *daf*-*21*(*RNAi*) reduces *daf*-*2*-induced lifespan extension (p < 0.001 compared to *daf*-*2*; *EV*). (**b**) DAF-21 is not necessary for the lifespan extension caused by the *daf*-*2*(*e1370*) mutation in the *daf*-*16*(*mgDf50*); *daf*-*16d/f::gfp* strain (p = 0.633). Lifespan assays were repeated three times. Survival curves were compared using the Kaplan-Meyer log rank test. Lifespan values are given in Supplementary Table S1. EV: empty vector RNAi.

Our findings identified DAF-21 as an isoform-specific modulator of DAF-16 activity. Further, we observed that a reduction in DAF-21 capacity from larval development limits *daf*-*2* induced longevity. Hence, we investigated how *daf*-*21*(*RNAi*) employed from hatching affects longevity specified by individual DAF-16A and D/F isoforms in the context of reduced ILS by using strains expressing single isoforms in a *daf*-*2*(*e1370*); *daf*-*16*(*mgDf50*) mutant background. In concordance with published data^20,21^ both *daf*-*16a::rfp* and *daf*-*16d/f::gfp* transgenic strains exhibited longer lifespan compared to *daf*-*2*(*e1370*); *daf*-*16*(*mgDf50*) background (Fig. 8a and b and Table S1) indicating that both isoforms are involved in *daf*-*2* induced longevity. *daf*-*21*(*RNAi*) further shortened the lifespan of *daf*-*2*(*e1370*); *daf*-*16*(*mgDf50*) double mutants suggesting DAF-21 might also target longevity promoting factors other than DAF-16. Importantly, *daf*-*21* knockdown consistently diminished the lifespan of the DAF-16A::RFP expressing strain in all four biological replicates, while in two out of four trials it failed to modify that of the DAF-16D/F::GFP transgenic worms (Fig. 8a,b and Table S1). These results indicate that an optimal DAF-21 capacity from larval development plays a role in longevity through selectively ensuring DAF-16A function.

## Discussion

**Figure 9:**
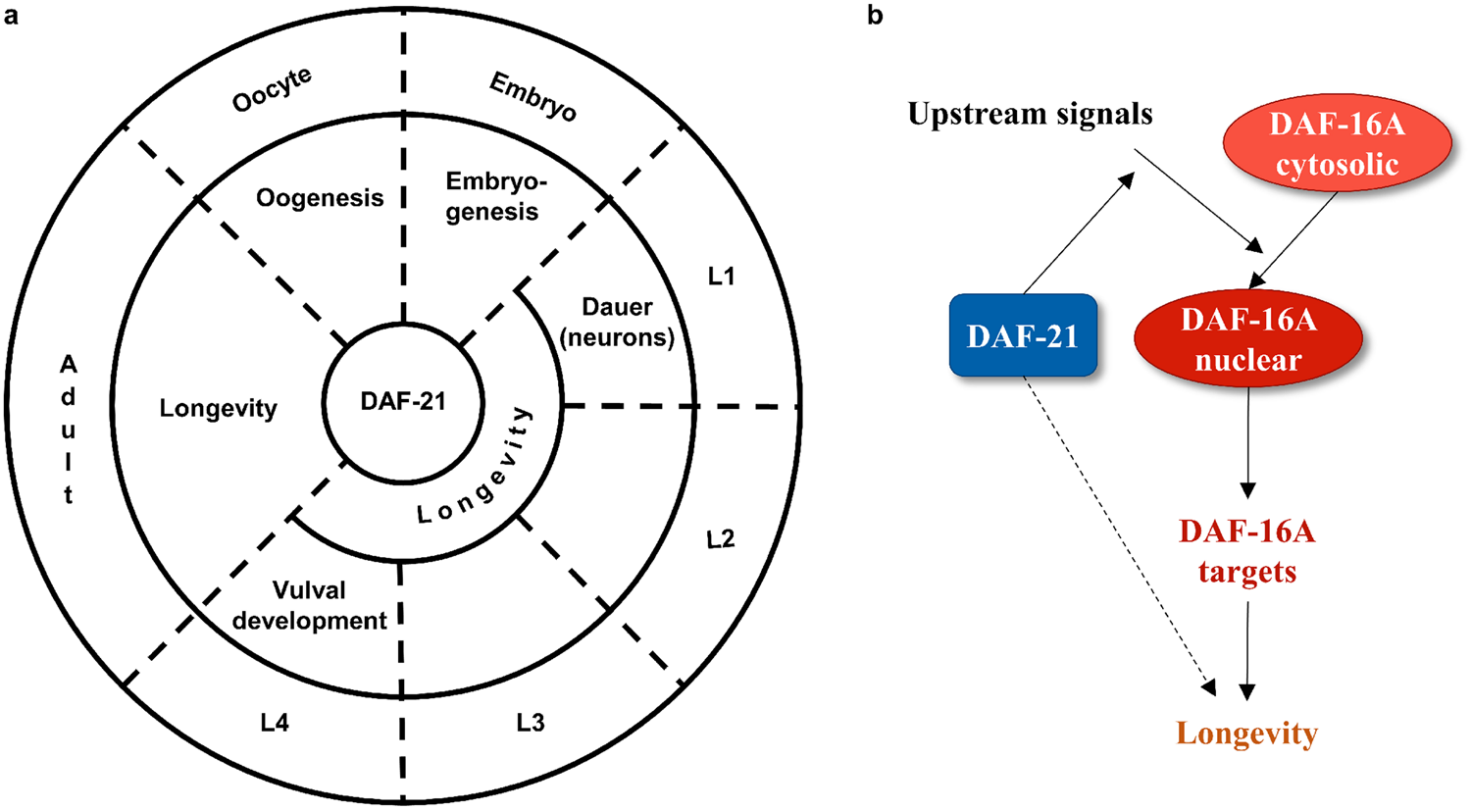
Proposed models depicting the regulatory roles of DAF-21/Hsp90 in *C*. *elegans*. (**a**) Spatiotemporal effects of DAF-21 during the nematode life cycle. Please note that DAF-21 is also required for motility^31^, which was not included in the figure. (**b**) Model of the effect of DAF-21 on DAF-16A activation and longevity. In response to upstream signals (such as reduced ILS or heat shock) DAF-21 promotes DAF-16A nuclear translocation and transcriptional function, which extends lifespan. DAF-21 also exhibits a DAF-16 independent effect on longevity (indicated by dashed line). For the sake of simplicity, we did not include the DAF-21 independent activation of DAF-16D/F in this model. See text for further details.

We have found that a reduction of DAF-21 capacity by RNA interference in non-neuronal tissues from the beginning of larval development shortens wild-type and extended lifespan conferred by lowered ILS. This effect persists in wild-type worms if RNAi treatment is initiated at the L4 stage and appears to operate both *via daf*-*16* dependent and independent routes. Lifespan reduction happens without apparent developmental problems, even despite an increased heat stress tolerance and decreased fertility. Our findings therefore propose a direct role for DAF-21/Hsp90 as a longevity regulator. We have also shown that dauer formation, in contrast, requires neuronal silencing of *daf*-*21*. Finally, we have observed that *daf*-*21* knockdown compromises oogenesis and embryonic development of the F1 generation. Thus, a spatiotemporal specificity governs the pleiotropic effects of DAF-21 to support fertility, larval development and longevity (Fig. 9). Such spatiotemporal function is also characteristic to other regulatory mechanisms. For instance, ILS during early larval development primarily affects dauer arrest, whereas during adulthood extends lifespan^44^. Likewise, neuronal *daf*-*16* regulates dauer development in larvae and intestinal *daf*-*16* is required for longevity during early adulthood^46^.

Our observations also suggest that the novel pro-longevity function of DAF-21 may be distinct from those controlling development although it already appears to operate during larval development. Though the identification of the mechanism(s) require further studies, potential candidates might be a less ordered organization of protein-protein and signaling networks^22^, a faster deterioration of proteostasis^59^ especially in muscle, where lack of DAF-21 impairs filamental structure integrity resulting in a decline in motility relative to wild type^31^. Likewise, an important aging-associated phenomenon is immune failure and bacterial infection, which is enhanced in *hsf*-*1*, *daf*-*16*, *skn*-*1* and also in *daf*-*21* nematodes^60–63^.

All the proteostatic defense, tissue integrity and immunity are more robust in *daf*-*2* mutants^19,60^, in which we have observed that *daf*-*21* silencing during adulthood does not shorten, but marginally extend lifespan. We speculate that the reduction of DAF-21 in early adulthood acting in concert with the mild metabolic stressor *daf*-*2* activates a pro-longevity response that antagonizes the *daf*-*21* induced lifespan shortening effect. Potential mechanisms might involve the HSF-1 dependent heat shock response^19,27^, the differential regulation of the respective DAF-16 isoforms^20,21^ (this study) or a *daf*-*21* independent pathway. However, the validation of this idea and the identification of the real mechanism(s) await further research.

Our findings provide evidence that DAF-21 contributes to longevity *via* specifically ensuring DAF-16A function, reflected by the specific requirement of DAF-21 for DAF-16A nuclear translocation, transcriptional function and lifespan extension mediated by DAF-16A as the sole DAF-16 isoform. Importantly, DAF-21 was necessary for the induction of three out of four DAF-16 target genes, each of them extends lifespan: *sod*-*3*^64^, *old*-*1*^65^, *scl*-*20*^52^ and *gst*-*20*^21^, the latter two identified as DAF-16A specific targets^21^ (Fig. 6a–d). *sod*-*3* has already been identified as a shared DAF-A and DAF-16D/F target^20,21^ and our findings propose *old*-*1* as a DAF-16A preferential target. In contrast, DAF-16D/F function was unaffected by *daf*-*21* knockdown. Although DAF-16B might also be influenced by DAF-21, its involvement in the DAF-21 dependent effects is highly unlikely, because it does not regulate lifespan and is mainly expressed in neurons and pharynx that are refractory to RNAi^20,49,51,66^.

This study confirms the involvement of both DAF-16A and D/F in lifespan control when ILS is lowered^20,21^. Our experiments using transgenes made by Kwon *et al*.^20^ do not permit us to draw a conclusion regarding the importance of the individual isoforms. A recent excellent study using single isoform mutants has shown that DAF-16D/F is only required for longevity when DAF-16A is not expressed and DAF-16A orchestrates a much more comprehensive gene expression pattern relative to DAF-16D/F (93% vs 30% of DAF-16 regulated mRNA-s)^21^. Based on this study, we propose a model: DAF-21 affecting the DAF-16A-regulated genes is required to elicit *daf*-*2*-induced *daf*-*16*-mediated longevity. If DAF-21 capacity is scarce, DAF-16A activity is compromised, DAF-16D/F may still evoke a compensatory response resulting in lifespan extension. Perhaps, this might underlie the lifespan extension and partial lifespan decrease, respectively, of *daf*-2 worms treated by *daf*-*21*(*RNAi*) in adulthood and from hatching. Other lifespan extending signals that selectively activate DAF-16D/F operate independently of DAF-21 availability.

What molecular mechanism(s) may be involved in the functional interaction between DAF-21 and DAF-16A? The fact, that all DAF-21, DAF-16A and DAF-16D/F are pleiotropically expressed in almost every tissue excludes the possibility of a merely tissue-specific interaction^20,31,59^. We observed a DAF-21 dependent DAF-16A nuclear translocation in muscle, hypodermis and intestine. Previous work identified that the intestinal, but not neuronal or muscle-specific expression of DAF-16 is responsible for the longevity effect of lowered ILS^46^. Furthermore, both muscle and intestinal DAF-16 combat reproductive aging^67^. Based on this, we speculate that the longevity promoting DAF-21-DAF-16A interaction may predominantly occur in the muscle and intestine.

Apart from tissue expression the isoform-specificity of the DAF-21-DAF-16A interaction may be due to differences in the respective sequences of the DAF-16A and D/F isoforms. It is established that the two isoforms share most of their 3′ exons including the DNA binding Forkhead domain, but the 5′ region corresponding to the N-terminal of the protein products are unique^2,20^ (Fig. 5a,b). A possible scenario might be that similarly to several transcription factors DAF-16A has an intrinsic instability which requires the chaperone function of DAF-21^22,23^. However, DAF-21 does not seem to stabilize DAF-16 because when *daf*-*21* was silenced, DAF-16A did not show aggregation, preserved its protein and mRNA level and biological function in the null mutant strain of *rle*-*1*, the E3 ubiquitin ligase responsible for the polyubiquitinylation of DAF-16^58^. Moreover, silencing *daf*-*21* did not prevent the nuclear import and transcriptional activity of the constitutively nuclear AKT-phosphorylation mutant DAF-16A^AM^::GFP^49^ indicating a proper folding of DAF-16A. These results also suggest that DAF-21 regulates DAF-16A activation upstream of its nuclear traffic. Promoter swapping between DAF-16A and D/F verified that the N-terminal segment of DAF-16A is responsible for the efficient nuclear entry of DAF-16^20^. Importantly, out of the three consensus AKT phosphorylation RxRxxS/T motifs conserved in human FOXOs and in DAF-16A and B, the N-terminal site of DAF-16D/F is replaced by a different QxRxxS which is probably the reason behind an asymmetrical regulation of the two isoforms by AKT-1 and AKT-2^20^. We speculate that the mechanism of the DAF-21-dependent differential interaction lies within the primary sequence of DAF-16 isoforms, subject to various regulatory modifications. One such isoform specific input has been published: TORC1 inhibition extended lifespan by inducing the nuclear translocation of DAF-16D/F without influencing DAF-16A^68^. Their and our findings lend support for an isoform-specific integration of distinct regulatory inputs by DAF-16/FOXO in longevity regulation. The biochemical nature of these inputs and the exact mechanisms of action is an important challenge for future studies.

DAF-21 is not the only heat shock protein that regulates DAF-16 nuclear traffic. HSF-1 and the constitutive HSP70 isoform HSP-1 are required for the nuclear export of DAF-16A/B in the *daf*-*16a/b::gfp* overexpressing TJ356 strain which, in turn, protects from the detrimental effect of DAF-16 hyperactivation on immunity^61^. Excessive activation of SKN-1/Nrf also led to compromised immune responses in *C*. *elegans*^62^ indicating a general importance to optimize the activity of stress inducible regulators. Likewise, an adequate orchestration of the activities of distinct master regulators are crucial for efficient organismal responses, such as the collaboration of HSF-1 with DAF-16 to extend lifespan in dampened ILS mutants^19^. Intriguingly, DAF-21/Hsp90 is a prime sensor of proteostasis, a stabilizer of signaling networks^22,26^ as well as a regulator of both stress-responsive transcription factors: it suppresses HSF-1^27,31,59^ while supports DAF-16A activation (this study). Thus, the reciprocal regulations through DAF-21 co-ordinating DAF-16 (and HSF-1) activity may ensure an integrated organismal response sensing both the steady state proteostatic milieu and nutrient availability, which promote proper self-maintenance and on the long term, longevity.

In summary, our work uncovers a previously unappreciated role of DAF-21 in *C*. *elegans* longevity which provides a crosstalk between the proteostasis and nutrient signaling networks *via* an isoform specific regulation of DAF-16 activity. Considering the strong structural and functional conservation of DAF-16/FOXO isoforms^3,18^ and DAF-21/Hsp90^22^, a similar regulatory mechanism might operate in mammals.

## Methods

### *C*. *elegans* strains and maintenance

All strains used were obtained from CGC. Animals were kept at 20 °C using standard *C*. *elegans* techniques^69^. *Caenorhabditis elegans* strains used in this study: N2: wild type, GR1307: *daf*-*16*(*mgDf50*), CF1038: *daf*-*16*(*mu86*), CB1370: *daf*-*2*(*e1370*); e1370/mu86: *daf*-*16*(*mu86*); *daf*-*2*(*e1370*), EFS7: *daf*-*16*(*mgDf50*); *daf*-*2*(*e1370*), TJ356: *daf*-*16p::daf*-*16a/b::gfp* + *rol*-*6*, HT1888: *daf*-*16*(*mgDf50*); *unc*-*119*(*ed3*); lpIs12 [*daf*-*16a::rfp* + *unc*-*119*(+)], HT1889: *daf*-*16*(*mgDf50*); *unc*-*119*(*ed3*); lpIs14 [*daf*-*16d/f::gfp* + *unc*-*119*(+)], HT1881: *daf*-*16*(*mgDf50*); *daf*-*2*(*e1370*); *unc*-*119*(*ed3*); lpIs12 [*daf*-*16a::rfp* + *unc*-*119*(+)], HT1883: *daf*-*16*(*mgDf50*); *daf*-*2*(*e1370*); *unc*-*119*(*ed3*); lpIs14 [*daf*-*16d/f::gfp* + *unc*-*119*(+)], HT1890: *daf*-*16*(*mgDf50*); *daf*-*2*(*e1370*), CF1371: [*daf*-*16*(*mu86*); *daf*-*16a*^*AM*^*::gfp/bKO* + *rol*-*6*(*su1006*)], JT6130: *daf*-*21*(*p673*), LL1009: daf-21(nr2081)/nT1 [unc-?(n754) let-?], KB6: *rle*-*1*(*cxTi510*), HT1888xKB6: *daf*-*16*(*mgDf50*); *unc*-*119*(*ed3*); lpIs12 [*daf*-*16a::rfp* + *unc*-*119*(+)]; *rle*-*1*(*cxTi510*), TU3335: *lin*-*15B*(*n744*) *X*; *uIs57 [unc*-*119p::YFP* + *unc*-*119p::sid*-*1* + *mec*-*6p::mec*-*6*].

### Crossing and genotyping

The *daf*-*2*(*e1370*); *daf*-*16*(*mu86*) double mutant strain was generated by crossing the CB1370[*daf*-*2*(*e1370*)] and CF1038[*daf*-*16*(*mu86*)] single mutants. Genotype of the F2 generation was monitored by allele-specific PCR (see Supplementary Table S7 for primer sequences).

The HT1888 and KB6 strains were crossed together and the *daf*-*16a::rfp* transgene was monitored by fluorescence microscopy in the F2 generation while the presence of *rle*-*1*(*cxTi510*) mutation was tested using allele-specific PCR.

### RNA interference

HT115(DE3) *E*. *coli* strains producing dsRNA against *daf*-*21* (*hsp90*)^35^ were kindly provided by Eileen Devaney (University of Glasgow, UK) and were created by cloning a 74 bp and a 294 bp region of *hsp90* into the L4440 vector. *daf*-*2*(*RNAi*)^70^ was from the Vellai Lab (Eötvös Loránd University, Budapest, Hungary) created by cloning a 1204 bp fragment of *daf*-*2* into the L4440 vector. All primers used can be found in Supplementary Table S7. RNAi treatment was performed using standard RNAi feeding method as described^71^: RNAi feeding *E*. *coli* clones were grown overnight in LB medium containing 100 μg/ml ampicillin. Worms were grown on plates containing 1 mM IPTG, 50 μg/ml ampicillin and 6.25 μg/ml tetracyclin and seeded with *E*. *coli* HT115 strains harboring the L4440 empty vector (EV) control and specific RNAi vectors, respectively, from hatching – or they were transferred onto RNAi plates after reaching the L4 larval stage on OP50. Measurements were made after 2 days on RNAi bacteria. To control for proper RNAi dosage in double RNAi treatments, overnight cultures of the RNAi bacterial strains were mixed in a 1:1 ratio, mixing in the empty vector harbouring strain in single RNAi controls.

### Lifespan assays

All lifespan assays were performed at 20 °C. Animals were synchronized by allowing gravid adults to lay eggs for 4 hours and using the next generation for experiments after they reached young adult stage. Approximately 35 animals were transferred to each of 3 plates containing 5-fluorodeoxyuridine (FUDR) (Sigma-Aldrich) to a final concentration of 51 µM. Day 1 is defined as the day the worms were placed on the FUDR plates. Every second day animals were scored by tapping with a platinum worm pick starting from day 7. Worms that crawled into the agar, onto the wall of the plate or died from vulval bursting were censored.

### Fluorescence microscopy

After treatments indicated in the figure legends at least 50 worms per condition were placed on a 2% agarose pad, and immobilized by adding 25 mM NaN_3_ in M9 buffer. Pictures were taken by a Leica DMI6000B epifluorescence microscope with a DFC480 camera (Figs 4 and S4) or by Nikon Eclipse E400 microscope with Diagnostic Instruments SPOT model 1.5.0 camera (Figs 5, S5 and S6) using GFP or RFP fluorescent filters, respectively. Epifluorescent microscopic images are representatives of at least 3 independent experiments. In the case of DAF-16 localization studies, animals were sorted into three categories: ‘nuclear’ refers to animals that showed exclusively nuclear localization, ‘intermediate’ labels animals that had both nuclear and cytosolic fluorescence and ‘cytosolic’ refers to animals with solely cytosolic GFP or RFP expression^72,73^. In case of RNAi treatment the animals were kept on RNAi feeding bacteria for two days after reaching L4 stage.

### mRNA expression analysis

mRNA from well-fed synchronized population of adult worms was isolated using GeneJET RNA Purification Kit (Thermo Scientific). The mRNA was then transcribed into cDNA by RevertAid™ Premium Reverse Transcriptase (Thermo Scientific). qPCR measurements were performed in an ABI 7300 Real-time PCR machine using Maxima™ SYBR Green/ROX qPCR Master Mix (Thermo Scientific). Primer sequences are listed in Supplementary Table S7. Relative amounts of mRNAs were determined using the Comparative Cycle Treshold Method for quantitation and normalized to beta-actin mRNA levels. Each experiment was repeated three times. In case of RNAi treatment the animals were kept on RNAi feeding bacteria for two days after reaching L4 stage.

### Thermotolerance assay

30 young adult animals from a synchronized population were transferred to each of 3 plates for every condition. Plates were put in an incubator preheated to 35 °C for 6 hours. Then, plates were placed back into a 20 °C incubator. Following a 5 hour recovery time the animals were scored every 24 hours by tapping with a platinum worm pick. Worms that crawled into the agar, onto the wall of the plate or died from vulval bursting were censored. All thermotolerance measurements were repeated at least three times. Statistical analysis was performed by comparing different conditions and using two-tailed Student’s T-test to determine the level of significance.

### Dauer assay

10 gravid hermaphrodites were allowed to lay eggs on each plate for 4 hours. Then the plates were transferred to 25 °C and animals were allowed to grow until the third day, when adults and dauer larvae were scored from each strain and condition.

### Phenotypic characterization

10 gravid hermaphrodites were allowed to lay eggs on EV and *daf-21(RNAi)* plates for 1 hour in order to get a highly synchronous population of animals. The plates were kept at 20 °C and progeny was scored for phenotypic differences after three days. Pictures were taken using Nikon Eclipse E400 microscope with Diagnostic Instruments SPOT model 1.5.0 camera.

### Fertility assay

10 L4 hermaphrodites from each condition of each strain were placed onto treatment plates individually. All plates were incubated at 20 °C. Every 24 hours mothers were transferred onto new plates. Progeny was scored on the plates 48 hours after the removal of the mother. The assay was carried on till the last animal stopped laying eggs.

### Western Blotting

Synchronized population of animals were grown on 10 cm NGM plates with IPTG seeded with either *daf-21*(*RNAi*) or empty vector (EV). Worms were washed three times using M9 buffer and frozen at −80 °C. After thawing, 200 µl of lysis buffer (50 mM This-HCl, 0.25% SDS, 1% IGEPAL CA-630, 150 mM NaCl, 1 mM EDTA, 2x Complete (Roche)) was added to the samples. After three freeze-thaw cycles samples were sonicated 6 times for 10 seconds and centrifuged for 10 minutes at 10000 g at 4 °C. Supernatant was transferred into new tubes and was stored at −20 °C or at −80 °C for longer storage. Western blotting was carried out as previously described^74^. Samples were run in 12% poly-acrylamide gel (Bio-Rad) and transferred to nitrocellulose membrane (Bio-Rad). Blocking was done by incubation in TBS-T with 5% skim milk powder for 1 hour at room temperature. Primary antibodies were Actin (monoclonal Anti-β-Actin, Sigma-Aldrich, 1:10000 in TBS-T with 5% BSA) and Hsp90^75^ (polyclonal Anti-Hsp90, 1:2000 in TBS-T with 5% BSA). Secondary antibodies were HRP-labelled anti-mouse (1:2000 in TBS-T with 5% skim milk powder) and anti-rabbit (1:3000) IgG, respectively (Dako). Membranes were incubated with ECL reagent (GE Healthcare) for 1 min and developed.

### Statistical analysis

Statistical analysis was done by the SPSS 15.0 software (SPSS Inc., Chicago, IL, USA). Survival curves were compared using the Kaplan-Meyer log rank test. Pairwise comparisons were done using Student’s t-test. Multiple comparisons were done with ANOVA using the Fisher’s Least Significant Difference (LSD) test. Variables were expressed as mean ± standard error of the mean (SEM). Statistical levels of significance are as follows: *p < 0.05, **p < 0.01, ***p < 0.001.

## Electronic supplementary material

Supplementary Information
