# Supplementary material for: DAF-21/Hsp90 is required for C. elegans longevity by ensuring DAF-16/FOXO isoform A function
Source: Sci Rep. 2018 Aug 13;8:12048. doi: 10.1038/s41598-018-30592-6 (PMC6089956; doi:10.1038/s41598-018-30592-6)
Supplement: Supplementary file 1 — Supplementary Information [file 41598_2018_30592_MOESM1_ESM.pdf]

DAF-21/Hsp90 is required for *C. elegans* longevity by ensuring DAF-16/FOXO isoform A function

Supplementary Information

Milán Somogyvári, Eszter Gecse & Csaba Sőti*

Department of Medical Chemistry, Semmelweis University, Budapest, Hungary

Content:

Supplementary Figures

Supplementary Tables

*Corresponding author. Department of Medical Chemistry, Semmelweis University, P.O.Box 2, Budapest, H-1428, Hungary. Tel.: + 36 1 4591500 extn. 60130; Fax: + 36 1 4591500 extn. 60141; E-mail: soti.csaba@med.semmelweis-univ.hu

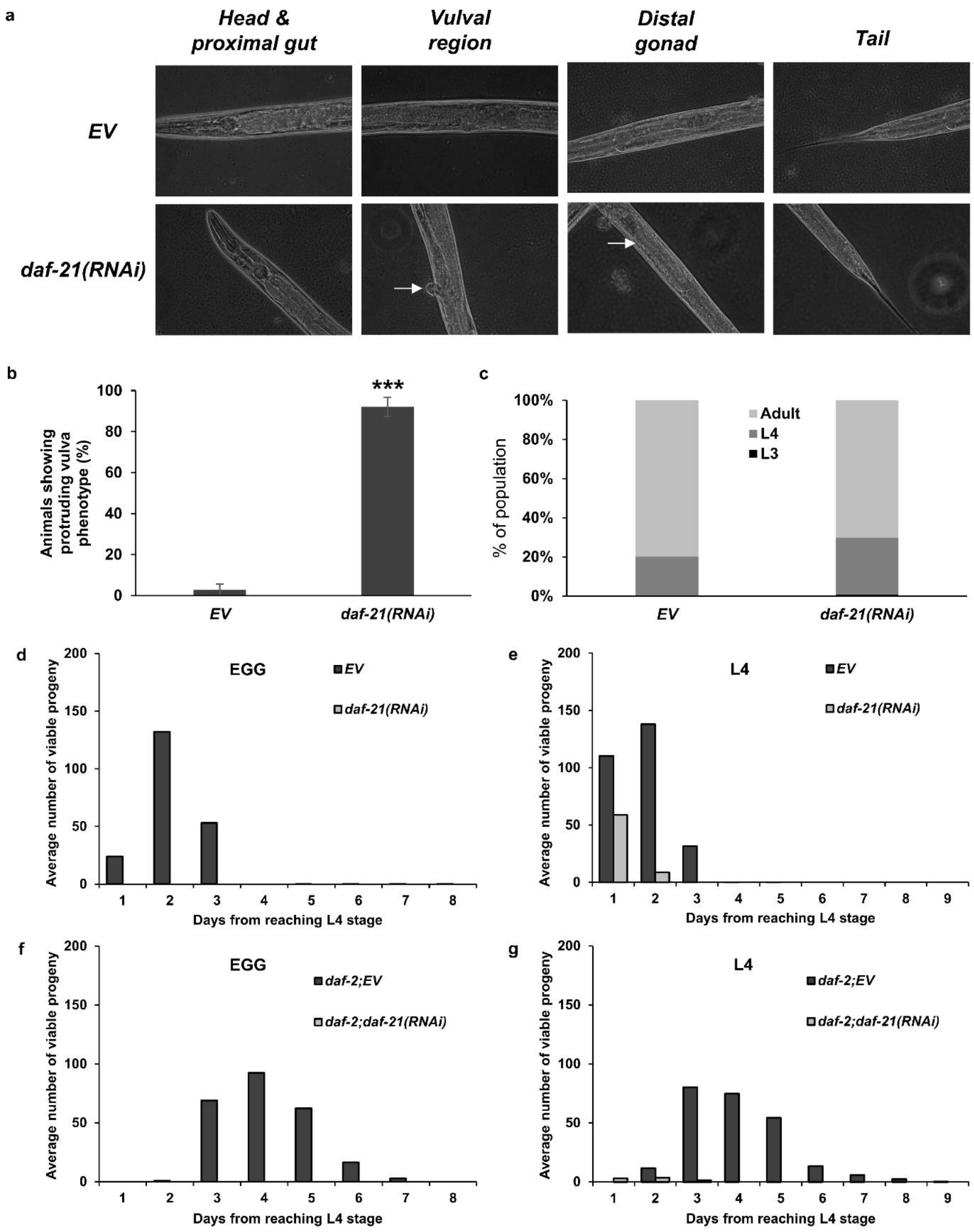
a
Head & proximal gut
Vulval region
Distal gonad
Tail
EV
daf-21(RNAi)
b
Animals showing protruding vulva phenotype (%)
100
80
60
40
20
0
***
EV
daf-21(RNAi)
c
% of population
100%
80%
60%
40%
20%
0%
Adult
L4
L3
EV
daf-21(RNAi)
d
EGG
EV
daf-21(RNAi)
Average number of viable progeny
200
150
100
50
0
1 2 3 4 5 6 7 8
Days from reaching L4 stage
e
L4
EV
daf-21(RNAi)
Average number of viable progeny
200
150
100
50
0
1 2 3 4 5 6 7 8 9
Days from reaching L4 stage
f
EGG
daf-2;EV
daf-2;daf-21(RNAi)
Average number of viable progeny
200
150
100
50
0
1 2 3 4 5 6 7 8
Days from reaching L4 stage
g
L4
daf-2;EV
daf-2;daf-21(RNAi)
Average number of viable progeny
200
150
100
50
0
1 2 3 4 5 6 7 8 9
Days from reaching L4 stage

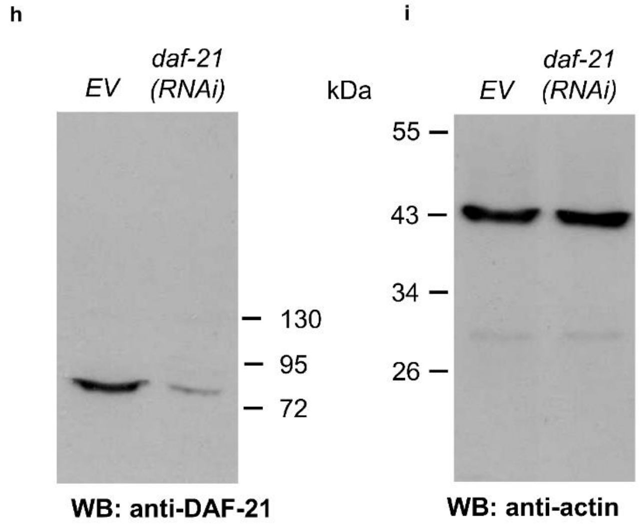

Supplementary Figure S1. Effect of DAF-21/Hsp90 knockdown on the development of wild-type and fertility of wild-type and *daf-2* mutant nematodes. *daf-21(RNAi)* causes protruding vulva phenotype (a, white arrow and b, p<0.001) and inhibits oocyte formation (a, white arrow) but no other apparent morphological abnormalities could be detected (a and c, p=0.123). Phenotypic data was analysed using Student's t-test for comparisons in SPSS 15.0 software. The bar graph represents the mean frequency of a phenotype while error bars show the Standard Error of Mean (SEM). *daf-21(RNAi)* employed from hatching (d and f; EGG) results in sterility, while that from the L4 stage (e and g; L4) reduces brood size in both N2 (d and e) and *daf-2(e1370)* (e and f) mutants. Fertility data are given in Supplementary Table S5. (h-i) Full length Western blot related to Fig. 1b.

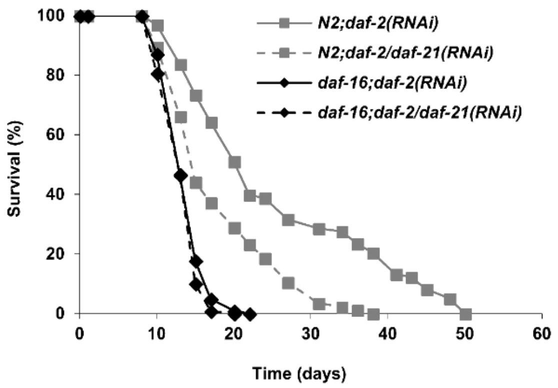

Supplementary Figure S2. Reduction of DAF-21/Hsp90 capacity limits *daf-2(RNAi)* induced longevity. *daf-21(RNAi)* reduces the extended lifespan of *daf-2(RNAi)* treated N2 worms ($p<0.001$) but does not change that of *daf-2(RNAi)* fed *daf-16(mu86)* mutants ($p=0.521$). RNAi treatments were employed from hatching and bacteria were mixed in a 1:1 ratio including the EV strain in single RNAi controls. Lifespan assays were repeated three times. Survival curves were compared using the Kaplan-Meyer log rank test. Lifespan values are given in Supplementary Table S1. EV: empty vector RNAi.

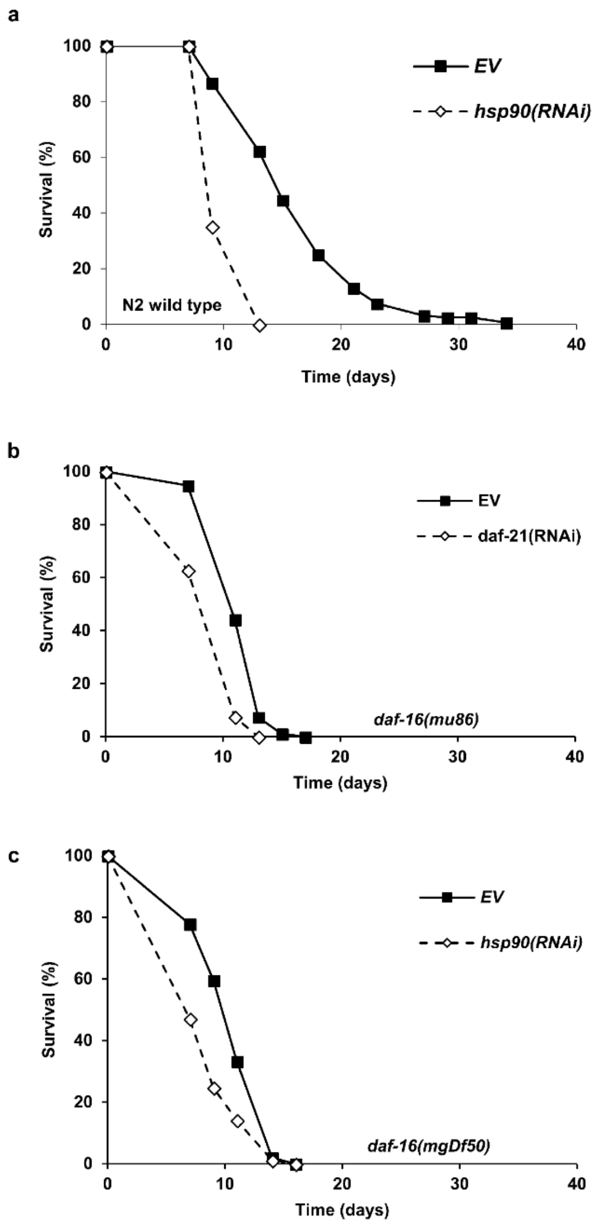

Supplementary Figure S3. DAF-21/Hsp90 knockdown also shortens lifespan in a *daf-16* independent manner. *daf-21(RNAi)* employed from hatching significantly reduces lifespan of wild-type worms (a) ($p<0.001$ compared to EV) and to a lesser, but still significant extent that of *daf-16(mu86)* (b) ($p<0.001$ in 3 out of 6 experiments) and *daf-16(mgDf50)* (c) ($p<0.001$) mutants. Lifespan assays were repeated three times. Survival curves were compared using the Kaplan-Meyer log rank test. Lifespan values are given in Supplementary Table S1. EV: empty vector RNAi.

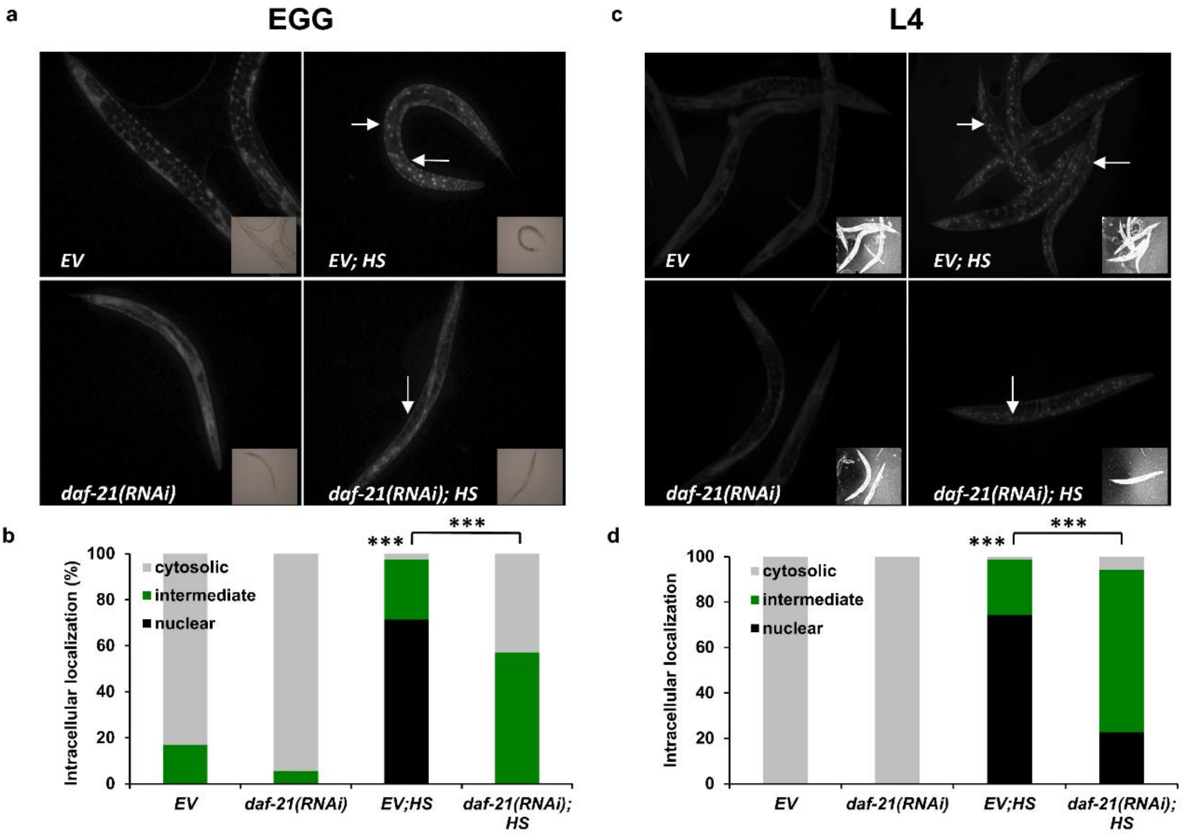

Supplementary Figure S4. DAF-21/Hsp90 is required for heat shock induced DAF-16A/B nuclear translocation. Representative epifluorescence microscopic images showing the heat shock (35°C, 1 hour) induced nuclear translocation of DAF-16A/B::GFP inhibited by *daf-21(RNAi)* treatment employed either from hatching (a) or from the L4 stage (c). White arrows indicate nuclearly localized GFP. Quantification (mean ± SEM) of DAF-16A/B::GFP localization from three independent experiments, each experiment using 30 animals per condition (b and d). Cytosolic refers to animals without nuclearly localized GFP signal, intermediate refers to animals with nuclear and cytosolic GFP and nuclear refers to animals with solely nuclear GFP signal. Microscopic images are representatives of 3 independent experiments. Statistics were analyzed by ANOVA and are given in Supplementary Table S4. HS: heat shock, EV: empty vector RNAi. ***:$p<0.001$.

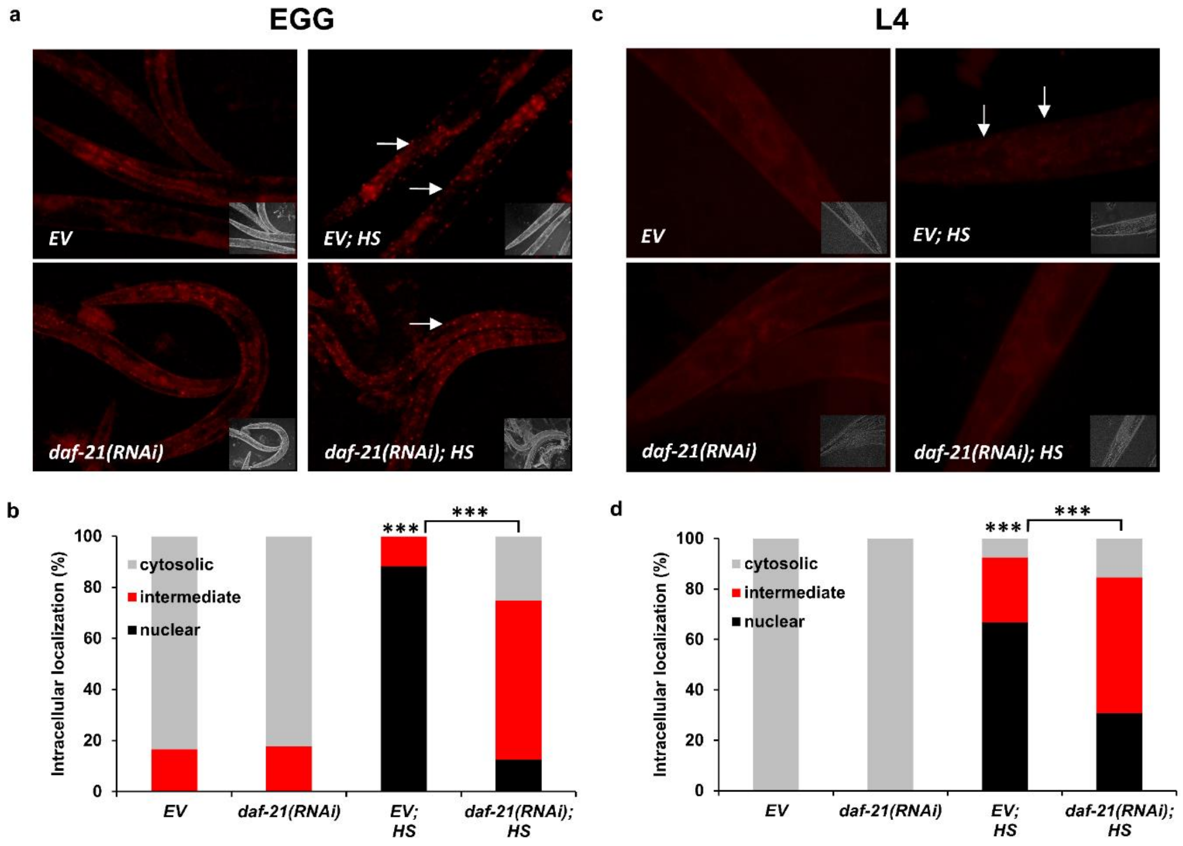

Supplementary Figure S5. DAF-16 isoform A requires DAF-21/Hsp90 for heat-shock induced nuclear translocation. Representative epifluorescence microscopic images showing the heat shock (35°C, 1 hour) induced nuclear translocation of DAF-16A::RFP inhibited by *daf-21(RNAi)* treatment employed either from hatching (a) or from the L4 stage (c). White arrows indicate nuclearly localized RFP. Quantification (mean ± SEM) of DAF-16A::RFP localization from three independent experiments, each experiment using 30 animals per condition (b and d). Cytosolic refers to animals without nuclearly localized RFP signal, intermediate refers to animals with nuclear and cytosolic RFP and nuclear refers to animals with solely nuclear RFP signal. Microscopic images are representatives of 3 independent experiments. Statistics were analyzed by ANOVA and are given in Supplementary Table S4. HS: heat shock, EV: empty vector RNAi. ***:$p<0.001$

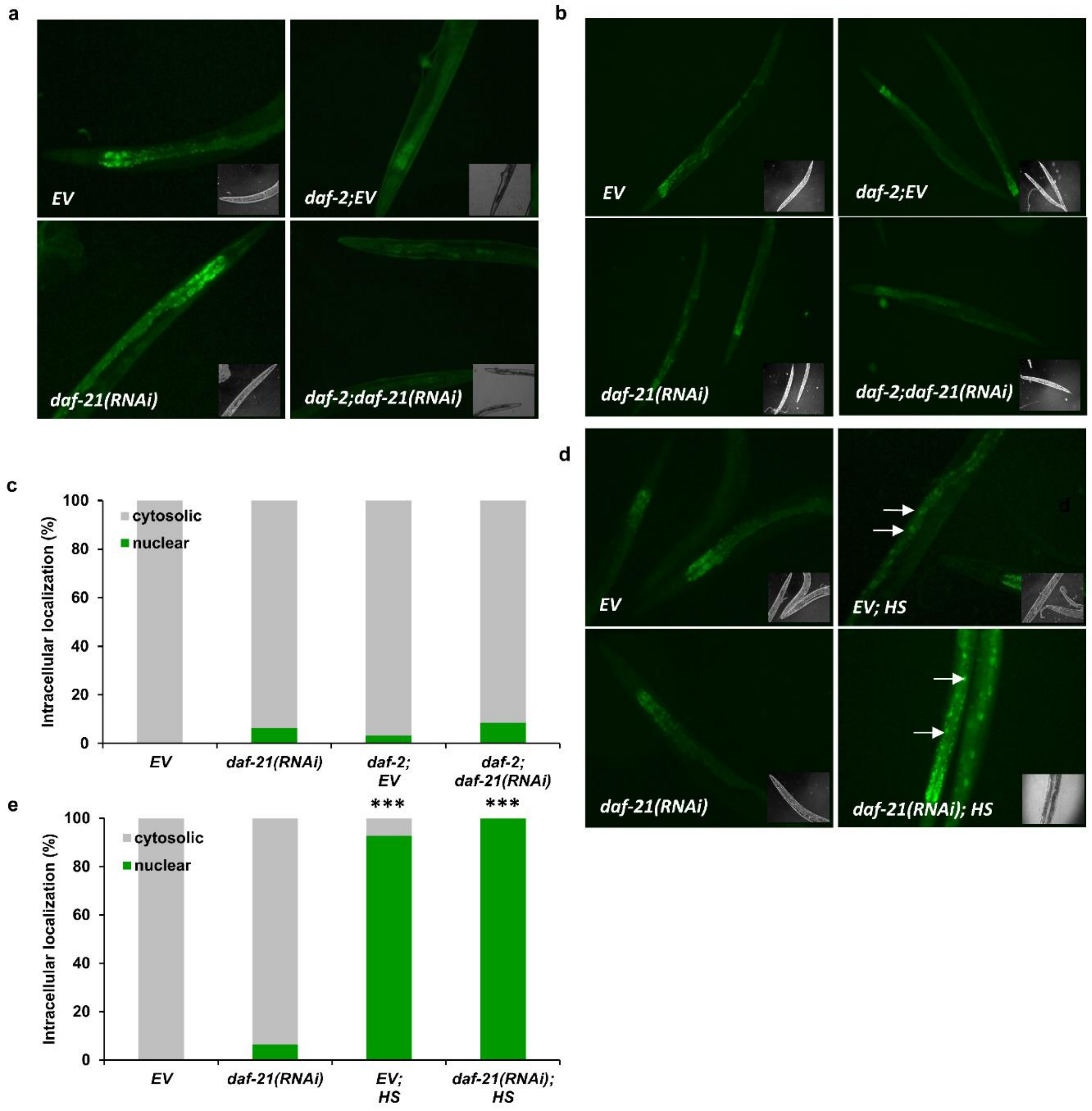

Supplementary Figure S6. *daf-21/hsp90* silencing does not influence the nuclear translocation of DAF-16D/F::GFP. The *daf-2(e1370)* mutant allele fails to induce nuclear localization of DAF-16D/F::GFP at 20°C (a) or at 25°C (b). (d) Heat-shock induced translocation of DAF-16D/F::GFP into the nucleus is not affected by *daf-21(RNAi)*. White arrows indicate nuclearly localized GFP. Please note the background autofluorescence in all samples. Quantification (mean ± SEM) of DAF-16D/F::GFP localization from three independent experiments, each experiment using 30 animals per condition (c and e). Cytosolic refers to animals without nuclearly localized GFP signal and nuclear refers to animals showing nuclear GFP signal. Microscopic images are representatives of 3 independent experiments. Statistics were analyzed by ANOVA and are given in Supplementary Table S4. HS: heat shock, EV: empty vector RNAi. ***:$p<0.001$

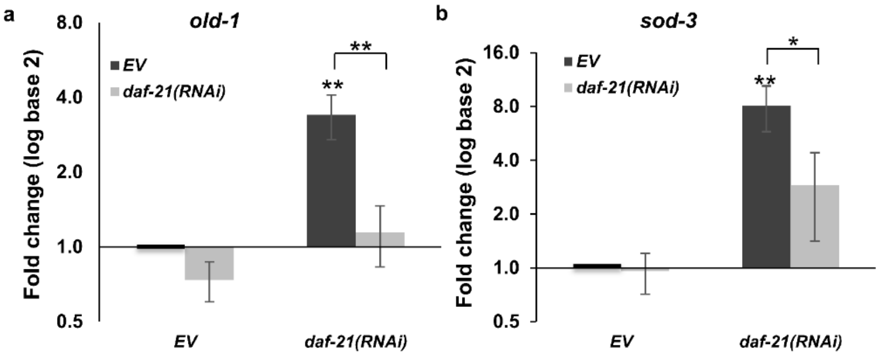

Supplementary Figure S7. DAF-21/Hsp90 is required for *daf-2(RNAi)* induced *old-1* and *sod-3* mRNA expression. Effect of *daf-21(RNAi)* and/or *daf-2(RNAi)* treatments on *old-1* (a) and *sod-3* (b) mRNA levels. RNAi treatments were employed from hatching. mRNA expression was assayed by qRT-PCR, normalized to *β-actin* mRNA and log2 transformed fold change values (mean ± SEM) were expressed relative to the respective EV control values of one. Data shown are from three independent experiments. qRT-PCR statistics were analyzed by ANOVA and are given in Supplementary Table S3. EV: empty vector RNAi.

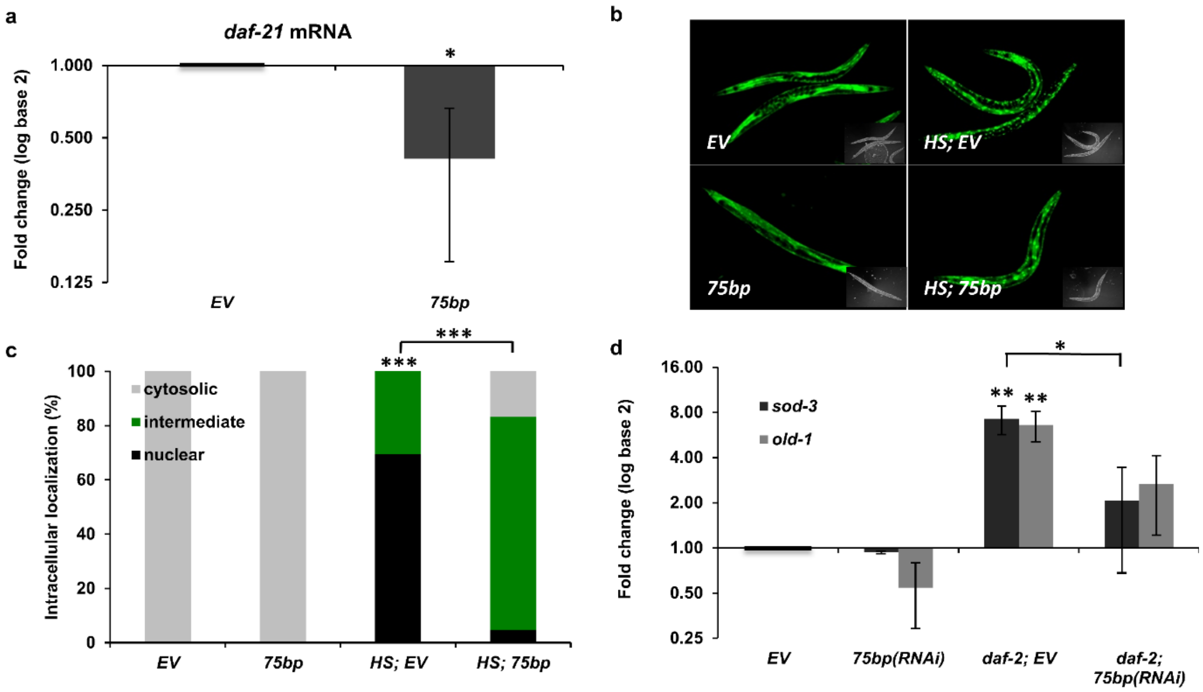

Supplementary Figure S8. Inhibition of DAF-16A activation by an independent *daf-21(RNAi)* construct. (a) The 75bp RNAi for DAF-21 reduces *daf-21* mRNA level. (b) Representative epifluorescence microscopic images showing the heat shock (1 hour at 35°C) induced nuclear translocation of DAF-16A/B::GFP inhibited by the 75bp *daf-21(RNAi)*. (c) Quantification of DAF-16A/B::GFP localization from three independent experiments, each experiment using 30 animals per condition. Cytosolic refers to animals without nuclearly localized GFP signal, intermediate refers to animals with nuclear and cytosolic GFP and nuclear refers to animals with solely nuclear GFP signal. Localization data was analysed by ANOVA and is expressed as the mean (± SEM) rate of animals with a particular localization phenotype. Statistical data are given in Supplementary Table S4. (d) The 75 bp *daf-21(RNAi)* inhibits the *daf-2* induced expression of *sod-3* and *old-1* mRNA-s, respectively, in the *daf-16a::rfp* transgenic strain. Single isoform transgenic *daf-16(mgDf50);daf-16a::rfp* worms and the *daf-2(e1370)* derivative were fed by EV or 75 bp *daf-21(RNAi)* from hatching. mRNA expression was assayed by qRT-PCR, normalized to *β-actin* mRNA and log2 transformed fold change values (mean ± SEM) were expressed relative to the respective EV control values of one. Data shown are from three independent experiments. qRT-PCR statistics were analyzed by ANOVA and are given in Supplementary Table S3. EV: empty vector RNAi. 75bp: 75 bp *daf-21(RNAi)*. *: $p<0.05$, ***: $p<0.001$.

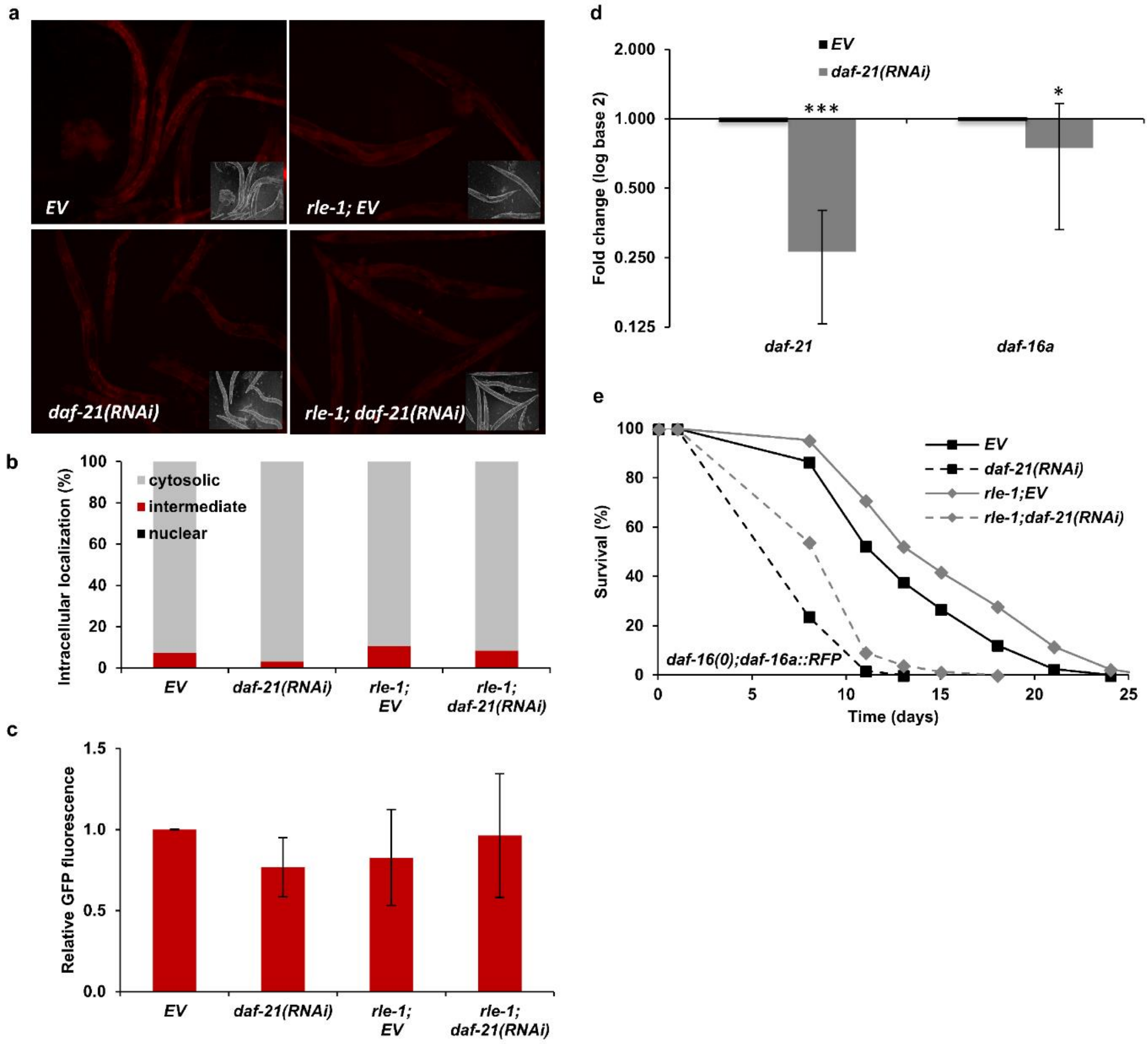

Supplementary Figure S9. DAF-21/Hsp90 knockdown neither destabilizes DAF-16A::RFP nor prevents lifespan extension in the *rle-1* ubiquitin ligase mutant. (a-c) *daf-21(RNAi)* does not affect the level and aggregation of DAF-16A::RFP in *rle-1* mutants. (a) Representative epifluorescence microscopic images showing DAF-16A::RFP signal in wild-type and *rle-1* backgrounds. Quantification of DAF-16A::RFP localization (b) and protein level (c) from panel (a) using data from three independent experiments, each experiment employing 30 animals per condition. Cytosolic refers to animals without nuclearly localized RFP signal, intermediate refers to animals with nuclear and cytosolic RFP and nuclear refers to animals with solely nuclear RFP signal. Localization data was analysed by ANOVA and is expressed as the mean (± SEM) rate of animals with a particular localization phenotype. Statistical data are given in Supplementary Table S4. (d) Effect of *daf-21(RNAi)* on *daf-21* and *daf-16a* mRNA levels. mRNA expression was assayed by qRT-PCR, normalized to *β-actin* mRNA and log2 transformed fold change values (mean ± SEM) were expressed relative to the respective EV control values of one. Data shown are from three independent experiments. qRT-PCR statistics

were analyzed by ANOVA and are given in Supplementary Table S3. *:$p<0.05$ ***: $p<0.001$. (e) The increased lifespan of *daf-16a::rfp* worms in *rle-1(cxTi510)* background ($p<0.01$ in 2 out of 3 trials compared to *daf-16a::rfp;EV*) persists upon *daf-21(RNAi)* treatment ($p<0.01$ in 2 out of 3 trials compared to *daf-16a::rfp;daf-21(RNAi)*). Lifespan assays were repeated three times. Survival curves were compared using the Kaplan-Meyer log rank test. Lifespan values are given in Supplementary Table S1. EV: empty vector RNAi.

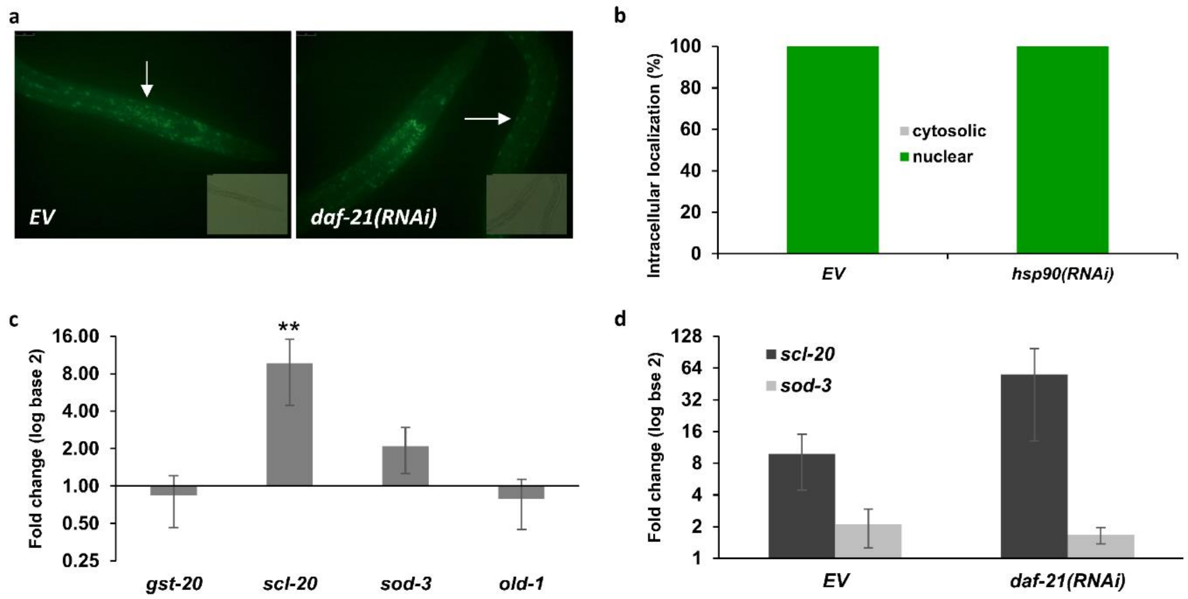

Supplementary Figure S10. DAF-21 is neither required for the nuclear localization of DAF-16A^{AM} nor for its transcriptional activity. (a) Nuclear localization of DAF-16A^{AM}::GFP in L4 animals fed by EV or *daf-21(RNAi)*. White arrows indicate nuclearly localized GFP. Epifluorescence microscopic images are representatives of 3 independent experiments. (b) Quantification of DAF-16A^{AM}::GFP localization from panel (a). (c) Expression of DAF-16A-specific target genes in DAF-16A^{AM}::GFP worms. Of four mRNA-s, only *scl-20* (p=0.006) shows significantly increased expression when compared to the *daf-16(mu86)* background strain while *sod-3* expression is also somewhat induced (p=0.351). (d) *daf-21(RNAi)* did not reduce *scl-20* or *sod-3* expression (p=0.374 and p=0.853, respectively). RNAi treatments were employed from hatching. mRNA expression was assayed by qRT-PCR, normalized to *β-actin* mRNA and log2 transformed fold change values (mean ± SEM) were expressed relative to the respective EV control values of one. Data shown are from three independent experiments. qRT-PCR statistics were analyzed by ANOVA and are given in Supplementary Table S3. EV: empty vector RNAi.

Table S1. Survival data of different nematode strains on empty vector (*EV*) and *daf-21(RNAi)*

**N2 wild type; Fig. 2 a and b**			**N Total (Dead)**	**Mean**	**% to *EV***	**Median**	**% to *EV***	**p vs. *EV***
Treatment from hatching	1	*EV*	79 (77)	19.5		19.0		
		daf-21(RNAi)	73 (70)	13.2	-32.1	12.0	-36.8	<0.001
	2	*EV*	166 (159)	18.0		16.0		
		daf-21(RNAi)	57 (57)	11.4	-36.8	10.0	-37.5	<0.001
	3	*EV*	73 (73)	15.7		15.0		
		daf-21(RNAi)	76 (75)	13.7	-12.8	13.0	-13.3	0.003
Treatment from L4	1	*EV*	104 (98)	18.1		17.0		
		daf-21(RNAi)	97 (91)	14.1	-22.1	13.0	-23.5	<0.001
	2	*EV*	110 (102)	17.6		17.0		
		daf-21(RNAi)	101 (77)	15.4	-12.4	13.0	-23.5	0.001
***daf-2(e1370)*; Fig. 2c and d**			**N Total (Dead)**	**Mean**	**% to *EV***	**Median**	**% to *EV***	**p vs. *EV***
Treatment from hatching	1	*EV*	44 (38)	27.3		26.0		
		daf-21(RNAi)	53 (50)	21.1	-22.5	19.0	-26.9	0.002
	2	*EV*	43 (43)	23.0		22.0		
		daf-21(RNAi)	28 (28)	16.6	-27.9	16.0	-27.3	0.001
	3	*EV*	93 (92)	23.7		24.0		
		daf-21(RNAi)	82 (61)	20.6	-13.0	19.0	-20.8	0.007
Treatment from L4	1	*EV*	89 (74)	26.4		24.0		
		daf-21(RNAi)	52 (46)	29.3	+11.0	17.0	-29.2	0.355
	2	*EV*	90 (76)	24.7		24.0		
		daf-21(RNAi)	116 (100)	41.2	+67.0	38.0	58.3	<0.001
	3	*EV*	53 (44)	21.2		20.0		
		daf-21(RNAi)	73 (71)	27.5	+29.4	20.0	0.0	0.076
	4	*EV*	46 (46)	17.1		13.0		
		daf-21(RNAi)	71 (67)	27.6	+61.4	22.0	69.2	<0.001
***daf-16(mgDf50)*; Fig. 2 a and b**			**N Total (Dead)**	**Mean**	**% to *EV***	**Median**	**% to *EV***	**p vs. *EV***
Treatment from hatching	1	*EV*	99 (99)	14.0		15.0		
		daf-21(RNAi)	43 (43)	10.5	-25.2	8.0	-46.7	<0.001
	2	*EV*	74 (72)	12.9		12.0		
		daf-21(RNAi)	56 (44)	12.3	-4.6	12.0	0.0	0.237
	3	*EV*	119 (113)	15.3		16.0		
		daf-21(RNAi)	55 (54)	12.6	-17.7	12.0	-25.0	<0.001
	4	*EV*	132 (132)	17.1		18.0		
		daf-21(RNAi)	128 (128)	15.8	-7.5	15.0	-16.7	<0.001
	5	*EV*	69 (65)	13.0		14.0		
		daf-21(RNAi)	78 (78)	12.5	-3.5	11.0	-21.4	0.054
	6	*EV*	78 (77)	12.9		12.0		
		daf-21(RNAi)	73 (73)	11.7	-9.5	10.0	-16.7	0.002
Treatment from L4	1	*EV*	82 (74)	14.0		13.0		
		daf-21(RNAi)	80 (72)	12.7	-9.8	13.0	0.0	<0.001
	2	*EV*	102 (66)	15.5		15.0		
		daf-21(RNAi)	84 (68)	13.3	-14.7	13.0	-13.3	<0.001
***daf-2(e1370);daf-16(mgDf50)*; Fig. 2c and d, 8a and b**			**N Total (Dead)**	**Mean**	**% to *EV***	**Median**	**% to *EV***	**p vs. *EV***
Treatment from hatching	1	*EV*	83 (83)	14.9		15.0		
		daf-21(RNAi)	77 (77)	11.8	-20.8	12.0	-20.0	<0.001
	2	*EV*	56 (56)	15.2		14.0		
		daf-21(RNAi)	30 (30)	12.4	-18.4	12.0	-14.3	<0.001
	3	*EV*	107 (106)	13.6		12.0		
		daf-21(RNAi)	60 (60)	11.4	-16.2	12.0	0.0	<0.001
	4	*EV*	113 (113)	16.8		18.0		
		daf-21(RNAi)	88 (88)	14.3	-14.9	15.0	-16.7	<0.001

	5	*EV*	83 (75)	14.7		14.0		
		daf-21(RNAi)	86 (86)	12.1	-17.9	11.0	-21.4	<0.001
	6	*EV*	68 (65)	13.8		15.0		
		daf-21(RNAi)	59 (59)	12.2	-11.8	12.0	-20.0	<0.001
	7	*EV*	84 (81)	12.8		12.0		
		daf-21(RNAi)	62 (60)	12.0	-6.8	10.0	-16.7	0.253
Treatment from L4	1	*EV*	67 (64)	13.2		13.0		
		daf-21(RNAi)	43 (41)	12.2	-7.2	13.0	0.0	0.020
	2	*EV*	82 (66)	13.3		13.0		
		daf-21(RNAi)	55 (42)	13.2	-0.4	13.0	0.0	0.890
	3	*EV*	95 (88)	14.6		13.0		
		daf-21(RNAi)	100 (97)	13.9	-4.4	13.0	0.0	0.178
	4	*EV*	113 (110)	15.5		15.0		
		daf-21(RNAi)	105 (104)	15.2	-2.2	15.0	0.0	0.435
***daf-2(e1370);daf-16(mgDf50);daf-16a::rfp;* Fig 8a**			**N Total (Dead)**	**Mean**	**% to *EV***	**Median**	**% to *EV***	**p vs. *EV***
Treatment from hatching	1	*EV*	60 (57)	18.5		15.0		
		daf-21(RNAi)	53 (53)	12.1	-34.7	10.0	-33.3	<0.001
	2	*EV*	99 (79)	19.3		16.0		
		daf-21(RNAi)	76 (70)	14.2	-26.2	14.0	-12.5	<0.001
	3	*EV*	77 (74)	24.4		24.0		
		daf-21(RNAi)	62 (57)	19.0	-22.4	19.0	-20.8	<0.001
	4	*EV*	85 (81)	23.7		24.0		
		daf-21(RNAi)	78 (72)	18.5	-21.8	17.0	-29.2	<0.001
***daf-2(e1370);daf-16(mgDf50);daf-16d/f::gfp;* Fig 8b**			**N Total (Dead)**	**Mean**	**% to *EV***	**Median**	**% to *EV***	**p vs. *EV***
Treatment from hatching	1	*EV*	105 (104)	41.8		44.0		
		daf-21(RNAi)	126 (120)	41.0	-2.0	42.0	-4.5	0.633
	2	*EV*	102 (102)	41.9		44.0		
		daf-21(RNAi)	93 (92)	26.2	-37.5	25.0	-43.2	<0.001
	3	*EV*	60 (60)	33.6		33.0		
		daf-21(RNAi)	49 (41)	28.4	-15.6	29.0	-12.1	0.004
	4	*EV*	84 (78)	27.4		26.0		
		daf-21(RNAi)	114 (101)	25.6	-6.7	24.0	-7.7	0.430

**RNAi treatment lifespan; Fig. S2**			**N Total (Dead)**	**Mean**	**% to *EV/daf-2(RNAi)***	**Median**	**% to *EV***	**% to *EV/daf-2(RNAi)***
1	*N2*	*EV/daf-2(RNAi)*	88 (67)	28.2		27.0		
		daf-2/daf-21(RNAi)	86 (83)	22.7	-19.3	20.0	+25.9	<0.001
	daf-16 (mu86)	*EV/daf-2(RNAi)*	86 (80)	14.6		15.0		
		daf-2/daf-21(RNAi)	83 (81)	14.4	-1.4	15.0	0.0	0.510
2	*N2*	*EV/daf-2(RNAi)*	110 (98)	26.7		22.0		
		daf-2/daf-21(RNAi)	87 (86)	18.3	-31.4	15.0	-31.8	<0.001
	daf-16 (mu86)	*EV/daf-2(RNAi)*	101 (101)	14.1		13.0		
		daf-2/daf-21(RNAi)	115 (109)	13.8	-2.1	13.0	0.0	0.521
3	*N2*	*EV/daf-2(RNAi)*	104 (90)	33.7		33.0		
		daf-2/daf-21(RNAi)	100 (98)	16.0	-52.5	16.0	-51.5	<0.001
	daf-16 (mu86)	*EV/daf-2(RNAi)*	107 (107)	13.0		12.0		
		daf-2/daf-21(RNAi)	64 (64)	12.8	-1.5	12.0	0.0	<0.001

daf-16(mgDf50);daf-16a::rfp; Fig S9e			N Total (Dead)	Mean	% to *control*	Median	% to *control*	p vs. *control*
Treat-ment from hatching	1	*EV*	79 (77)	14.6		15.0		
		daf-21(RNAi)	75 (75)	10.8	-25.9	10.0	-33.3	<0.001
		rle-1(cxTi510);EV	94 (93)	17.8	+21.9	17.0	+13.3	<0.001
		rle-1(cxTi510);daf-21(RNAi)	69 (69)	11.7	+7.9	12.0	20.0	0.220
	2	*EV*	82 (82)	13.6		13.0		
		daf-21(RNAi)	59 (59)	8.7	-35.9	8.0	-38.5	<0.001
		rle-1(cxTi510);EV	87 (86)	15.9	+16.7	15.0	+15.4	0.001
		rle-1(cxTi510);daf-21(RNAi)	76 (76)	9.9	+13.4	11.0	+37.5	<0.001
	3	*EV*	84 (84)	17.0		15.0		
		daf-21(RNAi)	70 (70)	12.0	-29.4	10.0	-33.3	<0.001
		rle-1(cxTi510);EV	94 (94)	18.6	+9.0	17.0	+13.3	0.072
		rle-1(cxTi510);daf-21(RNAi)	86 (85)	14.3	+19.0	12.0	+20.0	0.003

Table S2. : Statistical analysis of thermotolerance assays (t-tests of timepoints)

N2 wild type; Fig. 1g			Mean	% to *EV*	Median	% to *EV*	p vs. *EV*
Treatment from hatching	1	*EV*	44,9		35,0		
		daf-21(RNAi)	55,8	+24.4	59,0	+68.6	<0.001
	2	*EV*	20,9		11,0		
		daf-21(RNAi)	26,5	+26.8	11,0	0.0	0.026
	3	*EV*	37,4		35,0		
		daf-21(RNAi)	40,1	+7.1	35,0	0.0	0.520

Table S3. : Expression data from RT-PCR assays

N2 wild type, *daf-21* mRNA levels; Fig. 1a		Mean	% to *EV*	p vs. *EV*
Treatment from hatching	*EV*	1.0		
	daf-21(RNAi)	0.3	-70.0	0.036
Treatment from L4	*EV*	1.0		
	daf-21(RNAi)	0.3	-70.0	0.001

N2 wild type; Fig. 1c-f		*hsp-70* mRNA levels	p vs. *EV*	p vs. *daf-21(RNAi)*	*hsp-16.2* mRNA levels	p vs. *EV*	p vs. *daf-21(RNAi)*
Treatment from hatching	*EV*	1.0			1.0		
	EV/hsf-1(RNAi)	0.8	0.499		0.7	0.276	
	EV/daf-21(RNAi)	2.7	0.049		3.5	0.011	
	hsf-1/daf-21(RNAi)	0.7	0.367	0.020	1.3	0.608	0.018
Treatment from L4	*EV*	1.0			1.0		
	EV/hsf-1(RNAi)	0.4	0.027		0.6	0.162	
	EV/daf-21(RNAi)	2.6	0.027		2.0	0.010	
	hsf-1/daf-21(RNAi)	0.4	0.040	0.003	0.5	0.062	0.009

daf-16(mgDf50), treatment from hatching; Fig. 6a-d			Mean mRNA levels	p vs. *EV*	*p vs. daf-2* (e1370)
daf-16a::rfp	***sod-3***	*EV*	1.0		
		daf-21(RNAi)	0.5	0.017	
		daf-2(e1370);EV	2.1	0.014	
		daf-2(e1370);daf-21(RNAi)	1.2	0.492	0.043
	old-1	*EV*	1.0		
		daf-21(RNAi)	0.4	0.070	
		daf-2(e1370);EV	1.5	0.387	
		daf-2(e1370);daf-21(RNAi)	0.4	0.018	0.005
	scl-20	*EV*	1.0		

		daf-21(RNAi)	7.0	0.003	
		daf-2(e1370);EV	345.4	<0.001	
		daf-2(e1370);daf-21(RNAi)	166.2	<0.001	0.084
	gst-20	*EV*	1.0		
		daf-21(RNAi)	0.7	0.209	
		daf-2(e1370);EV	3.0	0.009	
		daf-2(e1370);daf-21(RNAi)	1.3	0.739	0.015
***daf-16(mgDf50)*, treatment from hatching; Fig. 6e-h**			**Mean mRNA levels**	**p vs. *EV***	***p vs. daf-2* (e1370)**
daf-16d/f::gfp	***sod-3***	*EV*	1.0		
		daf-21(RNAi)	1.6	0.943	
		daf-2(e1370);EV	4.5	0.272	
		daf-2(e1370);daf-21(RNAi)	5.6	0.216	0.873
	old-1	*EV*	1.0		
		daf-21(RNAi)	0.8	0.453	
		daf-2(e1370);EV	0.4	0.186	
		daf-2(e1370);daf-21(RNAi)	0.6	0.296	0.752
	scl-1	*EV*	1.0		
		daf-21(RNAi)	20.6	0.104	
		daf-2(e1370);EV	70.0	0.016	
		daf-2(e1370);daf-21(RNAi)	289.2	0.003	0.290
	lea-1	*EV*	1.0		
		daf-21(RNAi)	3.2	0.340	
		daf-2(e1370);EV	17.1	0.150	
		daf-2(e1370);daf-21(RNAi)	2.5	0.449	0.449

***daf-2(e1370)*, treatment from L4; Fig. 7a-d**		**Mean mRNA levels**	**p vs. *daf-2; daf-16;EV***	**p vs. daf-2*;EV***
sod-3	*daf-16(mgDf50);EV*	1.0		
	daf-16(mgDf50);daf-21(RNAi)	1.9	0.226	
	EV	78.7	<0.001	
	daf-21(RNAi)	24.9	<0.004	0.037
old-1	*daf-16(mgDf50);EV*	1.0		
	daf-16(mgDf50);daf-21(RNAi)	2.1	0.088	
	EV	9.9	<0.001	
	daf-21(RNAi)	3.6	0.012	0.028
scl-20	*daf-16(mgDf50);EV*	1.0		
	daf-16(mgDf50);daf-21(RNAi)	1.7	0.761	
	EV	5317.1	<0.001	
	daf-21(RNAi)	1949.7	0.001	0.390
gst-20	*daf-16(mgDf50);EV*	1.0		
	daf-16(mgDf50);daf-21(RNAi)	0.8	0.422	
	EV	4.4	0.002	
	daf-21(RNAi)	1.7	0.177	0.015
***daf-2(e1370)*, treatment from L4; Fig. 7e-h**		**Mean mRNA levels**	**p vs. daf-2; *daf-16;EV***	**p vs. *daf-2;EV***
scl-1	*daf-16(mgDf50);EV*	1.0		
	daf-16(mgDf50);daf-21(RNAi)	1.0	0.925	
	EV	2453.4	<0.001	
	daf-21(RNAi)	2305.2	<0.001	0.938
lea-1	*daf-16(mgDf50);EV*	1.0		
	daf-16(mgDf50);daf-21(RNAi)	2.4	0.010	
	EV	10.1	<0.001	
	daf-21(RNAi)	20.6	<0.001	0.027

col-183	*daf-16(mgDf50);EV*	1.0		
	daf-16(mgDf50);daf-21(RNAi)	2.4	0.198	
	EV	16.2	0.001	
	daf-21(RNAi)	22.1	0.001	0.860
R05D8.7	*daf-16(mgDf50);EV*	1.0		
	daf-16(mgDf50);daf-21(RNAi)	1.1	0.913	
	EV	14.4	0.006	
	daf-21(RNAi)	13.5	0.013	0.638

**N2 wild type, treatment from hatching; Fig. S7**		**Mean mRNA levels**	**p vs. *EV***	**p vs. *daf-2 (RNAi)***
old-1	*EV*	1.0		
	EV/daf-21(RNAi)	0.7	0.242	
	EV/daf-2(RNAi)	3.4	0.002	
	daf-2/daf-21(RNAi)	1.1	0.815	0.003
sod-3	*EV*	1.0		
	EV/daf-21(RNAi)	1.0	0.852	
	EV/daf-2(RNAi)	8.1	0.005	
	daf-2/daf-21(RNAi)	2.9	0.213	0.038

**N2 wild type; Fig. S8a**		***daf-21* mRNA levels**	**% to *EV***	**p vs. *EV***
Treatment from hatching	*EV*	1.0		
	75pb(RNAi)	0.3	-65.3	0.017

**Treatment from hatching; Fig. S8d**		**Mean mRNA levels**	**% to *EV***	**p vs. *EV***	**p vs. *daf-2;EV***
sod-3 mRNA levels	*N2;EV*	1.0			
	N2;75bp(RNAi)	0.9	-6.5	0.868	
	daf-2(e1370); EV	7.3	+625.2	0.007	
	daf-2(e1370);75bp(RNAi)	2.1	-71.6	0.191	0.022
old-1 mRNA levels	*N2;EV*	1.0			
	N;75bp(RNAi)	0.5	-45.9	0.162	
	daf-2(e1370); EV	6.6	+559.7	0.009	
	daf-2(e1370);75bp(RNAi)	2.7	-59.7	0.084	0.067

**N2 wild type, treatment from hatching; Fig. S9d**		**Mean**	**% to *EV***	**p vs. *EV***
daf-21 mRNA levels	*EV*	1.0		
	daf-21(RNAi)	0.3	-73.5	<0.001
daf-16a mRNA levels	*EV*	1.0		
	daf-21(RNAi)	0.7	-25.2	0.045

***T*reatment from L4; Fig. S10**			**Mean mRNA levels**	**p vs. *daf-16 (mu86)***
daf-16(mu86)			1.0	
daf-16(mu86), daf-16a^{AM}::gfp		***gst-20***	0.8	0.551
		scl-20	9.8	0.006
		sod-3	2.1	0.351
		old-1	0.8	0.461
***T*reatment from L4; Fig. S10**			**Mean mRNA levels**	**p vs. *daf-16 (mu86)***
scl-20	*daf-16(mu86), daf-16a^{AM}::gfp*	*EV*	9.8	

		EV/daf-21(RNAi)	55.7	0.374
**sod-3**	*daf-16(mu86), daf-16a*^{AM}*::gfp*	*EV*	2.1	
		EV/daf-21(RNAi)	1.7	0.853

Table S4. : Statistical analysis of Fluorescence Microscopy assays

***daf-16ab::gfp*; Fig. 4a and b**		**Nuclear localization (%)**	**p vs. *EV***	**p vs. *EV/daf-2(RNAi)***
Treatment from hatching	*EV*	2.3		
	EV/daf-21(RNAi)	1.3	1.000	
	EV/daf-2(RNAi)	17.3	0.048	
	daf-2/daf-21(RNAi)	1.7	1.000	0.042
***daf-16ab::gfp*; Fig. 4c and d**		**Nuclear localization (%)**	**p vs. *EV***	**p vs. *EV/daf-2(RNAi)***
Treatment from L4	*EV*	0.0		
	EV/daf-21(RNAi)	0.0	1.000	
	EV/daf-2(RNAi)	81.2	<0.001	
	daf-2/daf-21(RNAi)	3.4	1.000	<0.001

***daf-16ab::gfp*; Fig. S4a and b**		**Nuclear localization (%)**	**p vs. EV**	**p vs. *HS;EV***
Treatment from hatching	*EV*	0.0		
	daf-21(RNAi)	0.0	1.000	
	HS;EV	71.3	<0.001	
	HS;daf-21(RNAi)	0.0	1.000	<0.001
***daf-16ab::gfp*, Fig. S4c and d**		**Nuclear localization (%)**	**p vs. *EV***	**p vs. *HS;EV***
Treatment from L4	*EV*	0.0		
	daf-21(RNAi)	0.0	1.000	
	HS;EV	74.1	<0.001	
	HS;daf-21(RNAi)	22.7	0.161	0.002

***daf-16a::rfp*; Fig. 5c and d**		**Nuclear localization (%)**	**p vs. *EV***	**p vs. *daf-2;EV***
Treatment from hatching	*EV*	0.0		
	daf-21(RNAi)	2.0	1.000	
	daf-2(e1370);EV	59.5	<0.001	
	daf-2(e1370);daf-21(RNAi)	9.6	1.000	0.002
***daf-16a::rfp*; Fig. 5e and f**		**Nuclear localization (%)**	**p vs. *EV***	**p vs. *daf-2;EV***
Treatment from L4	*EV*	0.0		
	daf-21(RNAi)	0.0	1.000	
	daf-2(e1370);EV	16.1	<0.001	
	daf-2(e1370);daf-21(RNAi)	8.0	0.065	<0.001

***daf-16a::rfp*; Fig. S5a and b**		**Nuclear localization (%)**	**p vs. *EV***	**p vs. *HS;EV***
Treatment from hatching	*EV*	0.0		
	daf-21(RNAi)	0.0	1.000	
	HS;EV	88.3	<0.001	
	HS;daf-21(RNAi)	12.5	0.834	<0.001

***daf-16a::rfp*; Fig. S5c and d**		**Nuclear localization (%)**	**p vs. *EV***	**p vs. *HS;EV***
Treatment from L4	*EV*	0.0		
	daf-21(RNAi)	0.0	1.000	
	HS;EV	66.7	<0.001	
	HS;daf-21(RNAi)	30.8	<0.001	<0.001

***daf-16d/f::gfp*; Fig. S6a and c**		**Nuclear localization (%)**	**p vs. *EV***	**p vs. *daf-2;EV***
Treatment from hatching	*EV*	0.0		
	daf-21(RNAi)	6.4	0.595	
	daf-2(e1370);EV	3.3	1.000	
	daf-2(e1370);daf-21(RNAi)	8.6	0.218	0.983
***daf-16d/f::gfp*; Fig. S6d and e**		**Nuclear localization (%)**	**p vs. *EV***	**p vs. *HS;EV***
Treatment from hatching	*EV*	0.0		
	daf-21(RNAi)	6.4	1.000	
	HS;EV	92.8	<0.001	
	HS;daf-21(RNAi)	100.0	<0.001	1.000

***daf-16a::rfp*; Fig. S8b and c**		**Nuclear localization (%)**	**% to *EV***	**p vs. *EV***
Treatment from hatching	*EV*	0.0		
	75bp(RNAi)	0.0	-	1.000
	HS;EV	69.4	-	<0.001
	HS;75bp(RNAi)	4.8	-93.1	<0.001

***daf-16a::rfp*; Fig. S9a and b**		**Intermediate localization (%)**	**% to *EV***	**p vs. *EV***
Treatment from hatching	*EV*	7.4		
	daf-21(RNAi)	3.0	-59.0	1.000
	rle-1(cxTi510);EV	10.4	40.6	1.000
	rle-1(cxTi510);daf-21(RNAi)	8.3	-20.0	1.000
***daf-16a::rfp*; Fig. S9a and c**		**Relative GFP fluorescence (%)**	**% to *EV***	**p vs. *EV***
Treatment from hatching	*EV*	1.0		
	daf-21(RNAi)	0.8	-23.2	1.000
	rle-1(cxTi510);EV	0.8	-17.3	1.000
	rle-1(cxTi510);daf-21(RNAi)	1.0	16.4	1.000

Table S5. : Statistical analysis of fertility assays

**Treatment from hatching; Fig. S1d and f**			**Average number of offspring**	**% to *EV***
1	*N2*	*EV*	188.7	
		daf-21(RNAi)	0.0	-100.0
	daf-2(e1370)	*EV*	146.0	
		daf-21(RNAi)	0.0	-100.0
2	*N2*	*EV*	209.6	
		daf-21(RNAi)	0.0	-100.0
	daf-2(e1370)	*EV*	243.7	
		daf-21(RNAi)	0.0	-100.0

3	*N2*	*EV*	264.7	
		daf-21(RNAi)	0.0	-100.0
	daf-2(e1370)	*EV*	164.0	
		daf-21(RNAi)	0.0	-100.0
**Treatment from L4; Fig. S2e and g**			**Average number of offspring**	**% to *EV***
1	*N2*	*EV*	269.8	
		daf-21(RNAi)	79.4	-70.6
	daf-2(e1370)	*EV*	261.8	
		daf-21(RNAi)	2.0	-99.2
2	*N2*	*EV*	280.0	
		daf-21(RNAi)	67.4	-75.9
	daf-2(e1370)	*EV*	174.8	
		daf-21(RNAi)	6.9	-96.1
3	*N2*	*EV*	228.5	
		daf-21(RNAi)	64.4	-71.8
	daf-2(e1370)	*EV*	236.4	
		daf-21(RNAi)	26.1	-89.0

Table S6. : Statistical analysis of dauer assays

**Treatment from hatching; Fig. 3a**		**Dauer formation (%)**	**p vs. *EV***	**p vs. daf-2(e1370)**
daf-16a::rfp; daf-2(e1370)	*EV*	96.0		1.000
	daf-21(RNAi)	95.6	1.000	1.000
daf-16d/f::gfp; daf-2(e1370)	*EV*	53.0		0.081
	daf-21(RNAi)	47.2	1.000	0.037
daf-2(e1370)	*EV*	98.4		
	daf-21(RNAi)	97.5	1.000	
daf-2(e1370); daf-16(mgDf50)	*EV*	0.0		<0.001
	daf-21(RNAi)	0.3	1.000	<0.001

**Treatment from hatching; Fig. 3b**		**Dauer formation (%)**	**p vs. *N2;EV***	**p vs. *EV***
N2 wild type	*EV*	0.0		
	daf-21(RNAi)	0.0		1.000
TU3335 (*unc-119p::sid-1*)	*EV*	6.8	0.019	
	daf-21(RNAi)	51.4	0.012	0.021

Table S7: Primers used in this study

Name	Sequence
daf-2wt_FW	5'-CCTCATCATTACTCAAACCAATATATGG-3'
daf-2mt_RV	5'-GTTACACTCGGTGCTCAGT-3'
daf-16FW	5'-ATTGTGTTCATTTGCCCCGC-3'
daf-16RV	5'-GAAGGGAGCCCATCAATGCTC-3'
rle-G5	5'-CAGAATGGGATACTCGTCGGATGCTCC-3'
rle-G3	5'-GTTGTTCACGTGGAGTACACTGGGGTTTGTC-3'
Tc5	5'-GGATCATCTGTAACTATCCTCTATCG-3'
S510	5'-CATGAGGATACCTCGATGC-3'
actin_FW	5'-ATCACCGCTCTTGCCCCATC-3'

actin_RV	5'-GGCCGGACTCGTCGTATTCTTG-3'
hsp90_FW	5'-TCAGTTCGGAGTCGGATTCT-3'
hsp90_RV	5'-CGACCTCTCCCTCCTTCTTC-3'
sod-3_FW	5'-CAAAGCTTGTTCAACCGGTTGC-3'
sod-3_RV	5'-CCTCGTGAAGTTTCTCCTCGATCTG-3'
old-1_FW	5'-TTCGCTGAGAAGAATTCCACGATC-3'
old-1RV	5'-GATCTGTTTGCCCGGAGTTCTC-3'
scl-1_FW	5’-CAATCAAGCATTGTGGATGC-3’
scl-1_RV	5’-GGAATCCACGACCATTTTCC-3’
scl-20_FW	5’-GTTCGCTGGATAAATATGCCC-3’
scl-20_RV	5’-ACTCTTGGTTCTTCCATCCG-3’
lea-1_FW	5’-ATGTAGAGAACAAAGCAGCAG-3’
lea-1_RV	5’-CCTTGTCCTTGGTCTTGTC-3’
gst-20_FW	5’-TTCTAGACAGCTCTTCGCC-3’
gst-20_RV	5’-TTTGGAGTCCCGAACTGAG-3’
col-183_FW	5’-CCTGGAAACGATGGACAACC-3’
col-183_RV	5’-GTCCTCCAGCAGATCCACTT-3’
R05D8.7_FW	5’-TGATGTTTTGGTGAACAATG-3’
R05D8.7_RV	5’-TTACGATCCGCCAGGAATAG-3’
daf-16a_FW	5’-CCTATTCGGATATCATTGCC-3’
daf-16a_RV	5’-GGATCGAGTTCTTCCATCCG-3’
daf-2(RNAi)_FW	5’-TTGGAAGCTCTCGGAACAACCAC-3’
daf-2(RNAi)_RV	5’-ATGAACGACGTTGAAGGAGAAGG-3’
hsp90(RNAi)75bp_FW	5’-CTGCTGTTCCATCATCG-3’
hsp90(RNAi)75bp_RV	5’-CTCCTCCATGCGGGAAG-3’
hsp90(RNAi)300bp_FW	5’-ATCAACCCAGACCACGCTATCATGAAG-3’
hsp90(RNAi)300bp_RV	5’-TTAGTCGACCTCCTCCATGCG-3’
